# Charge Mobility in Discotic Liquid Crystals

**DOI:** 10.3390/ijms22020877

**Published:** 2021-01-16

**Authors:** Roberto Termine, Attilio Golemme

**Affiliations:** LASCAMM CR-INSTM, CNR-NANOTEC SS di Rende, Dipartimento di Fisica, Università Della Calabria, 87036 Rende, Italy; attilio.golemme@unical.it

**Keywords:** discotic liquid crystals, charge mobility, organic semiconductors, charge transport, self-organization

## Abstract

Discotic (disk-shaped) molecules or molecular aggregates may form, within a certain temperature range, partially ordered phases, known as discotic liquid crystals, which have been extensively studied in the recent past. On the one hand, this interest was prompted by the fact that they represent models for testing energy and charge transport theories in organic materials. However, their long-range self-assembling properties, potential low cost, ease of processability with a variety of solvents and the relative ease of tailoring their properties via chemical synthesis, drove the attention of researchers also towards the exploitation of their semiconducting properties in organic electronic devices. This review covers recent research on the charge transport properties of discotic mesophases, starting with an introduction to their phase structure, followed by an overview of the models used to describe charge mobility in organic substances in general and in these systems in particular, and by the description of the techniques most commonly used to measure their charge mobility. The reader already familiar or not interested in such details can easily skip these sections and refer to the core section of this work, focusing on the most recent and significant results regarding charge mobility in discotic liquid crystals.

## 1. Introduction

The constantly growing demand for devices with novel features has prompted the search for materials with improved performances, on which devices are based. This is true for many different technologies, including the diverse areas of application of semiconductors. For several decades organic semiconductors have been the subject of many studies [1,2,3], sustained by confidence in the opinion that they might one day replace, or at least compete with, more traditional inorganic materials. Such a confidence is based on several properties of carbon-based semiconductors, including the relative ease of specific tailoring of their properties via chemical synthesis, the potential low cost when mass producing them, and the possibility of using wet processes during device fabrication.

Many experimental and theoretical studies of the physical properties related to charge transport have been carried out in organic semiconductors [4,5,6,7,8,9,10,11,12,13,14,15,16,17], in synergy with synthetic efforts aimed at providing the fabric on which models can be tested by way of accurate measurements. Charge mobility is probably the single most important parameter for many applications of semiconductors and it represents the touchstone for performance comparison among different compounds. Like most macroscopic properties, charge mobility depends heavily upon the (macro)molecular aggregation features, i.e., it is a phase property rather than a molecular one, although of course the specific molecular arrangement within a certain phase will in turn depend on the molecular structure. For this reason, one of the main research lines in the field of organic semiconductors has been the design of specific molecular structures that could, among other things, induce the formation of phases with molecular arrangements favouring charge transport [9].

The paramount role of order, both in the space and time domains, for achieving high charge mobility values, has been recognized since the first attempts at modeling charge transport in organic materials [18]. Although other factors do play a role, as a rule of thumb higher order translates into higher mobility. The length-scale of the uniform order must of course be at least comparable to the size of the device where a certain material is used, which varies widely. In general, especially when using wet and/or fast processes, which are desirable for mass production, organic crystals tend to form crystalline “powders”, where a homogeneous order is present only within each domain. Each grain boundary introduces disorder and it is a site where a carrier is slowed down or even trapped, severely affecting the observed overall charge mobility [19]. With some materials, it is so difficult to obtain ordered domains large enough to be tested in research labs, let alone attained on a production line, that the experimenters must contrive complex and often time-consuming protocols in order to obtain them [20].

For such a reason, it may be reasonable to compromise by losing some order while gaining processability. This may be accomplished by using liquid crystals, where the order of 3D solids is only partially lost, and for the same reason larger homogeneously ordered domains are generally achievable. In this context, thermotropic liquid crystals offer a variety of alternatives, with different degrees of translational and orientational order. Although other factors play a role, the formation of mesophases is heavily linked with the shape of the molecular units, in particular with their shape anisotropy, averaged by conformational motion. Thermotropic mesophases are usually formed by molecules with either a prolate (rod-like) or an oblate (disk-like) shape. Such anisotropic shapes induce anisotropic intermolecular interactions, often leading to long-range orientational order, which is the hallmark of liquid crystals.

**Figure 1:**
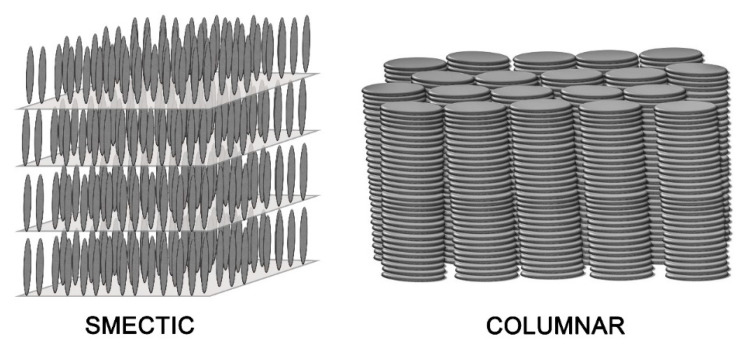
Schematic molecular organization in smectic A (formed by rod-like molecules) and columnar (formed by disc-like molecules) mesophases.

Charge transport in different kinds of mesophases has been the subject of many studies [21,22,23,24,25,26]. The nematic (N) phase is the least ordered mesophase, showing only orientational order (average molecules orientations along a common direction) and no long-range positional order. It can be considered a “translational liquid” and in most cases it does not exhibit semiconductor properties. Smectic phases, usually formed by molecules with a rod-like shape, exhibit a layered phase structure, as illustrated in Figure 1 in the case of the smectic A phase. This phase can be considered as a 1D solid, with positional order along the direction normal to the layers, while within the layers there is only orientational order. Other smectic phases, in addition to the layered structure, may exhibit further degrees of orientational and positional order within the layers: as the order increases, the structure gets closer and closer to the one of a 3D solid. In the most ordered smectic phases, charge mobilities up to 10^−2^–10^−1^·cm^2^·V^−1^·s^−1^ could be measured for charges moving in directions within the layer planes, but exceptional values of the order of 10 cm^2^·V^−1^·s^−1^ were also reported [27].

However, it was in the mesophases formed by molecules with a disk-like shape (see Figure 1) that charge transport showed the most consistent and promising results. Most thermotropic mesophases formed by oblate molecules are characterized by a columnar structure, with long-range orientational order within each column. In addition, different columns are organized to form a regular 2D lattice, so that these phases can be considered as 2D solids, as described in detail in the following section. In such columnar phases, high and highly anisotropic charge mobilities were measured, with the highest mobilities along the columnar axes.

The scope of this paper is limited to the review of charge mobility studies in thermotropic mesophases formed by molecules with a discotic shape. Synthetic aspects will not be covered and techniques for fabricating devices with long-range uniform domains [28,29,30,31,32,33,34] will only be given limited consideration. Although reference to some milestone results will be provided, this work does not intend to cover the subject from a historical perspective, with previously published reviews being widely available [35,36,37,38,39], but it will instead focus on the most recent advancements in the field. In Section 2 the different thermotropic mesophases formed by disk-shaped molecules will be described, while Section 3 will introduce the models used for understanding charge transport in organic materials and in liquid crystals in particular. Section 4 will cover the different techniques used for measuring charge mobility. Section 5 will give an account of the most recent and meaningful results, highlighting the potential of discotic mesophases as semiconductors.

## 2. Discotic Mesophases

**Figure 2:**
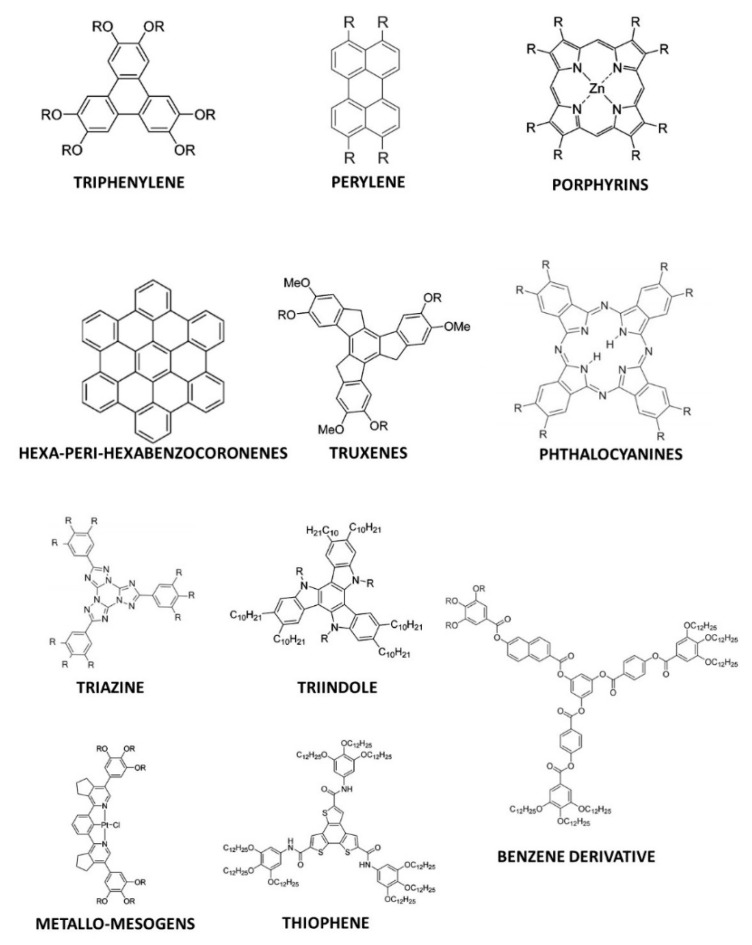
Structures of the most common cores of discotic mesogens.

In the context of this work, the term “discotic” mesophase will be used to indicate a liquid crystalline phase formed by organic molecules with a roughly disk-like shape. Usually, such molecules include a rather rigid, often planar or almost planar core: both the rigidity and the planarity are provided by a more or less extended π-electron system. Molecules with structures where such a rigid element is the only, or at least the main, component do not tend to form mesophases but rather well ordered, three-dimensional solids. However, the picture changes when flexible molecular elements are incorporated at the periphery of the structure. This is often accomplished by covalently linking alkyl chains to the core, either directly or via linking atoms such as oxygen or sulfur or groups such as alkyne, phenyl or ester moieties. In this case, thermodynamically stable mesophases may be formed. The particular degree of order and the dynamics, i.e., the phase nature, are determined not only by the core–core interactions, but also by the length, flexibility, branching and steric hindrance of the more flexible peripheral units. Figure 2 shows some of the most common molecular structures of the cores of discotic mesogens.

**Figure 3:**
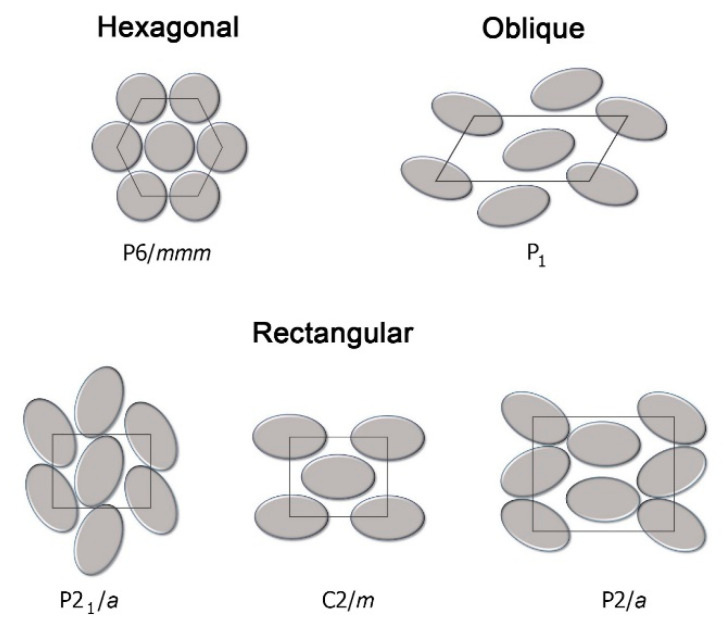
Different 2-D columnar organizations, observed within a cross-section normal to the direction of the columns, and their space groups. An elliptical cross-section indicates a tilt of the disks with respect to the column axis.

By far, the most common organization observed for such mesogens is the one with rigid cores assembled into stacks, forming parallel columns that arrange in space with regular 2-D patterns. In general, 2-D lattices are unstable [40] but in this case they are stabilized by the curvature elasticity (bending) of the columns [41] (p. 398). X-ray diffraction, both on monodomains and on powder samples, has been the most common experimental technique used to assess the nature of molecular order within such phases [42], although most investigations take advantage of contributions deriving from differential scanning calorimetry (DSC) and polarized optical microscopy (POM), while the role played by nuclear magnetic resonance (NMR) spectroscopy is also relevant [43,44,45]. Figure 3 contains illustrations of different columnar organizations, as observed within a cross-section normal to the direction of the columns. The rich polymorphism exhibited by columnar phases is generally classified according to the symmetry of the 2-D arrangement of the columns and to the average orientation of the flat cores with respect to the axes of the columns. There exist different classification schemes, i.e., the same phase may be named differently in different literature works. From a historic perspective, the first scheme was based on planar space groups [46,47,48], where the symmetry elements related to translations in the direction of the column axes are omitted. More recently, a classification based on IUPAC recommendations [49] became increasingly established. However, common practice nomenclature is abundant so that, for example, the columnar hexagonal phase, with a structure shown in Figure 3, may be named Col_h_, p6mm (or P6/mmm) or even D_h_ by different authors.

**Figure 4:**
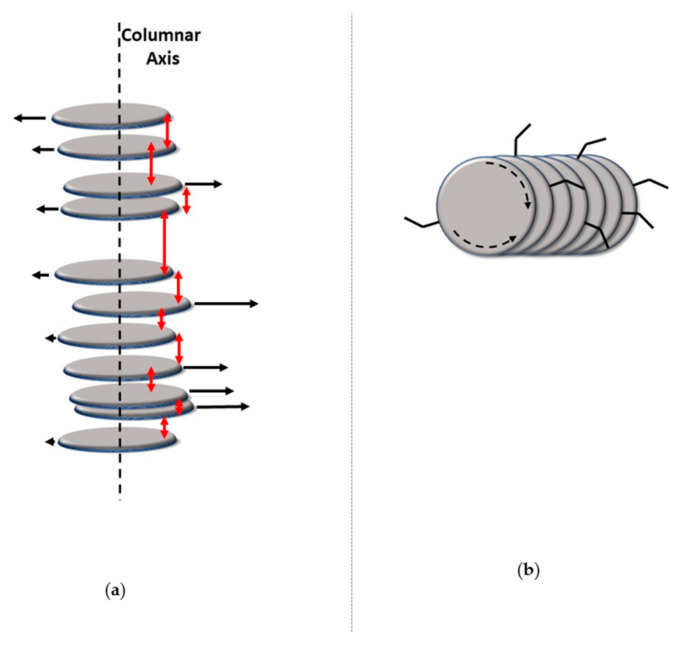
Schematic illustration of: (**a**) lateral and stacking disorder; (**b**) freedom of rotation around short disk axes.

In this phase, column axes are arranged in a hexagonal 2-D pattern and the short axes of the discoids that approximate the molecular shape are on average parallel to the column axes. In some cases, the same symmetry is achieved when such short axes are uniformly tilted with the same azimuthal angle within a column, but such an azimuthal angle does not show any long-range correlation among columns [50]. Like in all other columnar structures, in hexagonal columnar phases there exist a nano-segregation: the rigid sections of molecules are arranged within the columns, while the intercolumnar space is filled by the peripheral units, which exhibit a higher number of conformational degrees of freedom. In the Col_h_ phase, the lateral flexible chains are conformationally molten. The same 6-fold phase symmetry can coexist with different details of molecular order and dynamics. In the most disordered phase, often called disordered hexagonal columnar, cores do not have a regular long-range intracolumnar staking order but they can be considered as forming a 1-D liquid. In addition to such longitudinal (along the column axis) disorder, a certain degree of lateral disorder and freedom of rotation around short disk axes is also present, as illustrated in Figure 4. In other, more ordered, variants of the columnar hexagonal phase, there is a regular intracolumnar stacking distance between cores, but rotational freedom is preserved. In other cases, even rotational freedom is suppressed, often as a consequence of specific intermolecular interactions due to hydrogen bonding or to the presence of permanent electric dipoles [51,52].

More ordered, less symmetric columnar structures belong to the family of rectangular columnar phases Col_r_. In this case, cores are on average uniformly tilted within a columnar unit and there is a long-range intercolumnar correlation among the azimuthal angles. Such a correlation implies a greatly reduced conformational dynamics of the peripheral chains. An example of a cross section of columnar and molecular arrangements in Colr phases is illustrated in Figure 3, where the elliptical shapes symbolize tilted molecular cores. A less common columnar phase with an oblique 2-D lattice Col_obl_ is also known [53]. Some substances can exhibit both Col_h_ and Col_r_ phases, separated by a weak first-order transition.

This brief overview underlines how all details of molecular dynamics and orientational/positional order are not always captured by a “simple” description of phase symmetry. For example, phases where, in addition to the 2-D intercolumnar order, also a 1-D intracolumnar positional order is present, might be viewed as solids rather than mesophases. A lexical semantic discussion of this issue, related to the residual rotational dynamics of the whole molecule and to the degree of crystalline order in the peripheral chains, is outside the scope of this review. However, the details of positional and orientational order and of dynamics are of paramount importance in determining charge transport properties and they should always be considered in addition to a phase symmetry description, since the nano-segregated structure of columnar phases may induce subtle but important order/dynamics differences among phases of the same symmetry formed by different mesogens.

**Figure 5:**
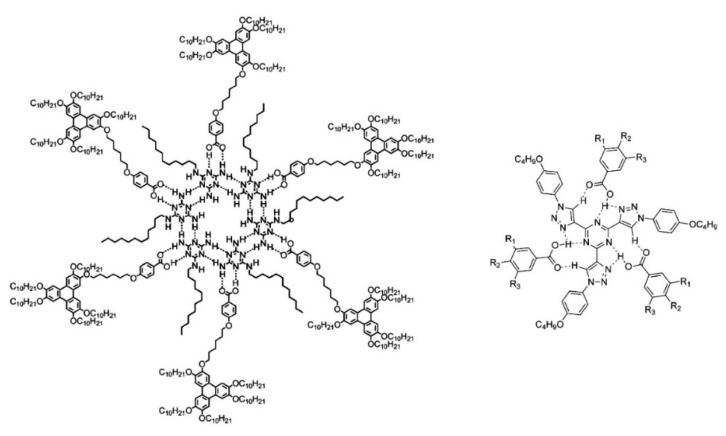
Some examples of molecular architectures and assemblies based on hydrogen bonds.

Beyond the flat core/flexible periphery paradigm, columnar phases can be obtained using a variety of other molecular architectures [54], such as conical cores [55,56], macrocycles [57,58,59] or metallomesogens [60,61]. In addition, the disk-shaped unit may not necessarily be a single entity of covalently linked atoms: in the recent literature there are in fact numerous examples of columnar phases formed by “supramolecular” disk-shaped units, where different dendrons assemble under driving forces tied to molecular shape and/or hydrogen bonds, dipolar or other specific interactions [62,63,64]. Figure 5 shows some examples of these molecular architectures and assemblies.

**Figure 6:**
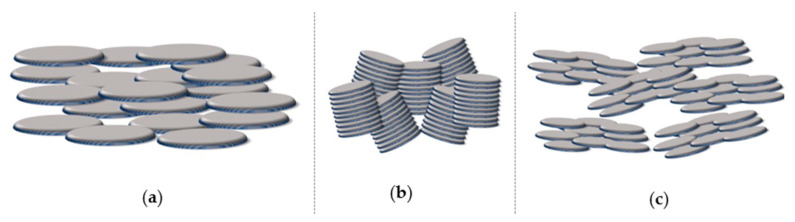
Nematic discotic phases: (**a**) nematic discotic, (**b**) nematic columnar, (**c**) nematic lateral.

The least ordered discotic mesophase is the nematic phase N_D_ [37]. In this phase, which is far less common than the columnar ones, there is only orientational order and no long-range translational order: molecules spontaneously orient with their disk normal along an average direction. The N_D_ phase shares the same symmetry with the nematic phase formed by rod-shaped molecules, and thus it shows the same schlieren textures under polarized microscope observations. Discotic nematics are diamagnetically and optically negative, while their dielectric anisotropy at low frequencies may have either sign, depending on the existence and orientation of permanent dipoles. As illustrated in Figure 6, there are a few variants of discotic nematic phases in which the building blocks are not single molecular entities but molecular aggregates. In the columnar nematic N_C_ there exist short columnar aggregates [65,66,67], so that while orientational order is long-range, positional order (along the columns) is only short-range. Similarly, in the lateral nematic N_L_ the aggregates are due to strong side interactions [68]. The evidence for the existence of such supramolecular structures derives mainly from POM and X-ray diffraction.

## 3. Charge Transport in Molecular Materials

The first studies regarding electronic applications of molecular materials date back to many decades ago, therefore it is to some extent surprising to realize how little we understand about the charge transport mechanisms in such semiconductors. Although progress is constant, the picture is not sharp even for molecular crystals, where the high degree of order simplifies modeling. Of the several physical parameters which are relevant to the use of molecular materials as semiconductors, the electrical conductivity σ obviously holds center stage. In isotropic materials, it can be defined as a function of the volume density ρ of mobile charge carriers and of charge mobility μ as:

σ = q·ρ·μ(1)

where q is the charge of the carrier. While in amorphous phases all the quantities in such a definition are scalars, in crystalline or mesomorphic phases both conductivity and mobility must be considered as second rank tensors. Our understanding of conductivity is then based upon how different models can account for a description of both ρ and μ.

The mere existence of a molecular entity implies a certain degree of localization of electrons within a molecule: the energies required to alter the electronic ground state, and thus promote the formation of mobile carriers, are much higher than thermal energy, even well above room temperature. This means that the density of thermally excited carriers, often referred to as intrinsic carriers, is usually very low. Charge density can increase due to the presence of defects, but in molecular materials the preferred method for increasing the number of mobile carriers is doping with atoms or molecules whose electronic energy levels promote electron transfer from/to the host material. In addition, mobile charge carriers can be introduced within the semiconductors by photogeneration or from electrodes, if injection barriers are minimized.

However, the main parameter limiting the application of molecular materials as semiconductors is generally identified in the charge mobility. Mobility can be viewed as measuring the macroscopic response of charge carriers, in terms of the rate of spatial displacement, to the stimulus provided by an electric field $\vec{E}$:

(2) $$\vec{v}=\;\mu \cdot \vec{E}$$

where $\vec{v}$ is the time-averaged velocity of carriers. Again, in anisotropic materials the mobility should be treated as a tensor. Given the fact that mobility is in general field dependent, a definition implying a linearization is often used, especially in the literature dealing with the comparison between experimental measurements and theoretical models, and thus mobility is defined as $\mu =\;\frac{\partial v}{\partial E}$.

Modelling charge transport in molecular substances is far from an easy task. In fact, strong interactions among atoms exist only on the length-scale of molecular dimensions, while intermolecular interactions are much weaker, being limited to dispersive forces, to those due to permanent dipoles and, when possible, to hydrogen or halogen bonding. In this context, even in the presence of crystalline or semi crystalline order, the density of static defects may be high and carrier transport may be determined by the grain boundary energetics rather than by molecular structure or packing [19,69]. In addition, even in the case of hypothetical defect-free structures, the weak interactions between molecules induce low frequency lattice vibrational modes that couple to the charge transport. In other words, the energetics of the electronic intermolecular interactions is quantitatively of the same order of magnitude as the energetics of the molecular/lattice deformations [70]. This often prevents the use of perturbative approaches in dealing with electron/phonon couplings in molecular semiconductors.

This scenario is often discussed in terms of two parameters, one related to the extent of electronic intermolecular interactions and the other one to the deformations introduced by the moving charge. The transfer integral t quantifies the degree of overlap of the molecular wavefunctions involved in the electron transfer between adjacent molecules. A small value of t implies a small tendency towards electron transfer. The transfer integral is obviously dependent upon the details of molecular structure, intermolecular distance and relative orientation, i.e., upon the packing [4]. Transfer integrals of the order of 100 meV are typical for high mobility, low molecular weight crystalline organic semiconductors [71], while in columnar phases formed by disk-shaped molecules they can be almost one order of magnitude higher in the best cases [72,73]. The reorganization energy λ accounts instead for the geometric relaxations associated with the gain/loss of an electron. It can be considered as deriving from two distinct contributions. The internal reorganization energy λ_in_ takes into account the variation of molecular geometry as a consequence of the charge loss or gain and it is strongly dependent upon molecular structure. The external reorganization energy λ_out_ considers the geometric relaxation associated with the variation of the polarization of the surrounding medium. Typical values for λ fall within the 50–500 meV range [4,12], with larger molecular structures usually showing values towards the lower end, due to their lower tendency to distort upon charge variations. The description of charge transport is highly simplified when only one of these two parameters, t or λ, plays a dominant role.

When t >> λ, the framework that best describes charge dynamics is the one of Bloch electrons with delocalized wavefunctions, moving as described by the semiclassical Boltzmann band transport model [74]. The positional order of molecules is reflected in the description of the carrier as a delocalized wavepacket, which is accelerated by an electric field between “collisions”. If for the time being we ignore nuclear motion, collisions are limited to those with lattice defects, which include impurities and surfaces. In this limiting case, considering an average time τ between collisions, a charge *e* will have a mobility:

(3) $$\mu =\frac{e\cdot \tau }{m^{*}}$$

In other words, the charge behaves as if it had an effective mass m*, which describes the ease of the propagation and that, in the simple case of one-dimensional systems, can be expressed as:

(4) $$m^{*}=\;\frac{{ћ}^{2}}{2\cdot t\cdot a^{2}}$$

where *a* is the separation in space between two lattice sites and *t* is the transfer integral. The picture is similar to that usually considered in the case of the classical inorganic semiconductors, except that for molecular materials intermolecular interactions are weak, which is another way of saying that transfer integrals are not so high: the lower charge mobility can then be interpreted in terms of a higher effective mass.

However, nuclei are never at rest, and this has consequences for charge transport. When an electron is added to or removed from a molecule, the equilibrium geometry changes, not only if one considers the structure of an isolated molecule but also in reference to the polarization of the surrounding medium. The ensemble of the charge and of the correlated deformations is called polaron [74]. If the charge is delocalized over many molecular entities, also the deformation is similarly distributed, so that charge transport can be seen as the propagation of a distributed charge carrying along a distributed deformation. Within this limit, not only the deformation of each single molecular entity is small due to delocalization, but reorganization is suppressed because charge mobility becomes so fast that the residence time of the charge on a particular site can become much smaller than the time required for nuclear motion. Considering this aspect related to nuclear motion, also the effect of temperature must be considered. As the temperature is raised, both intramolecular and intermolecular motions are excited, in particular lattice vibrations, which have low excitation energies due to the weak intermolecular interactions. The effect of a higher temperature is the decrease of the transfer integrals, thus increasing the effective mass of the carriers and decreasing mobility. On the basis of such considerations, the model of Boltzmann band transport can be used for molecular crystals at low temperatures and low level of defects and impurities. It is for such conditions that molecular materials usually reach high mobilities.

One can think of the effect on the transfer integrals of a progressively increasing temperature in terms of a progressive localization of the polaron. When the size of the polaron nears the distance between sites, the very concept of the electron as a delocalized Bloch-wave loses physical grounds [5,75]. In other words, the mean free path between collisions of the moving charge with thermal lattice vibrations becomes comparable to the molecular size. This is the scenario of the other limiting case, where t << λ. In this frame, the charge is localized on a molecular site and its movement is thermally activated, i.e., there is an energy barrier to be overcome for the charge to change site. This process is often termed hopping and it is equivalent to a redox chemical reaction involving the neutral molecule A and its ions, like the following one for electron hopping:

A + A^−^ ⇄ A^−^ + A (5)

or a similar one for hole hopping. In this case it is possible to extract electron transfer rates from transition state theory [76,77,78]. If, between consecutive hops between identical molecules, on average the charges spend enough time on each molecular site to allow for nuclear vibrational relaxation, there will be an associated free energy barrier for charge transfer and, in the classical limit *k_B_·T* >> ħω, one obtains for the transfer rate *k_ET_*:

(6) $$k_{ET}=\;\frac{t^{2}}{ћ}\;\sqrt{\frac{\pi }{k_{B}\cdot T\cdot \lambda }}\;e^{\frac{-\;}{4\cdot k_{B}\cdot T}}$$

where *k_B_* is the Boltzmann constant, *T* is the temperature and ω is a typical vibrational frequency of the system. The charge mobility can then be given by:

(7) $$\mu =\;\frac{a\cdot k_{ET}}{E}$$

**Figure 7:**
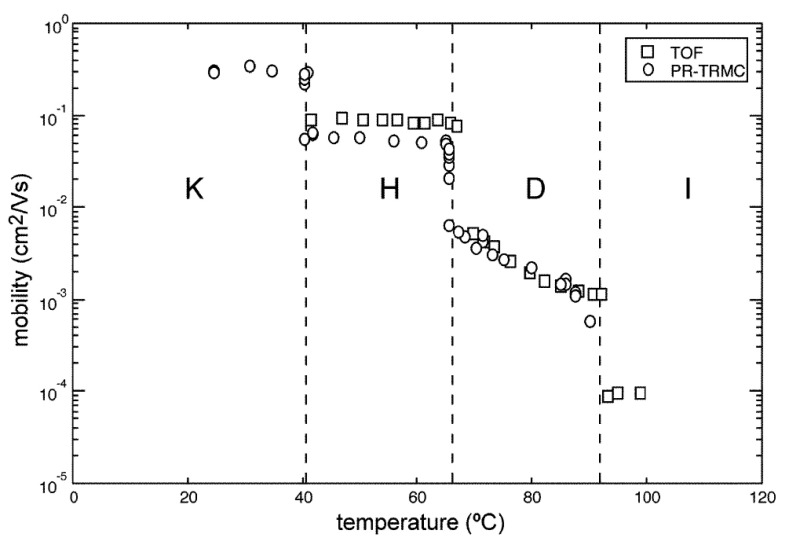
Temperature dependence of mobility in the different (K = crystal; H = helical columnar; D = hexagonal columnar; I = isotropic) phases of a triphenylene derivative. (Reprinted from reference [81] with permission from John Wiley and Sons. Copyright 2004 by John Wiley and Sons).

Up to this point, the issues related to the degree of molecular positional and orientational order, relevant to a discussion of charge transport, have only been hinted at, when describing the influence of temperature. Most models consider the distinct effects of two different types of disorder. One of them is the so-called diagonal disorder, a distribution of the energies of the frontier orbitals involved in charge transport. The origin of diagonal disorder can be traced back to conformational freedom and electrostatic and polarization interactions with neighboring molecules, especially in the presence of permanent dipoles. Besides being intuitively obvious, there is much experimental evidence that a higher degree of order is associated with better charge transport. It is known, for example, that in crystals formed by low molecular weight molecules, charge mobility can change by several orders of magnitude by just changing the preparation conditions of samples [79]. One of the best examples of the effect of order on mobility comes from the temperature dependence of hole mobility across the different phases formed by a discotic mesogen, a triphenylene derivative [80,81], as shown in Figure 7. It is evident how the mobility exhibits discrete jumps across phase transitions between phases characterized by different degrees of order. More extreme causes of disorder include impurities and grain boundaries. However, not all of them necessarily show a drastic negative effect on charge transport. This can be understood when considering the distribution of frontier orbital levels as a source of charge traps: when the energetic level at a particular site is much lower than in the surroundings, the charge residence time will increase and particularly deep levels become charge traps. However, not all impurities or vacancies necessarily create interband states and thus their effect on charge transport is not always ominous. In any case, in simulations energetic disorder is often described in terms of a Gaussian distribution of highest occupied molecular orbital (HOMO) or lowest unoccupied molecular orbital (LUMO) levels. Disorder can also influence charge transport via a different effect: in fact, not only the energy of a site can be affected, but also its interaction with neighboring sites. The variation of electronic coupling among molecules, due to variations of distances and relative orientations, is generally called off-diagonal disorder and it generates a distribution of transfer integrals.

The two limiting cases of band transport and hopping mentioned above have been used for decades to interpret experimental charge transport data in molecular materials. Band transport is suitable to describe ordered crystalline phases with a limited number of structural defects and impurities and at lower temperatures. The models that envisage a localized charge instead, are suitable to describe crystals at intermediate to higher temperatures, less ordered phases and materials with higher concentrations of impurities or defects, including grain boundaries. It is obvious that there are systems for which the conditions for a realistic application of neither of the limiting cases are satisfied. Several models have been proposed to describe charge transport in this regime, where t ~ λ. Among these, a relatively recent contribution to the field gives an interesting insight based upon the consideration of the dynamics of off-diagonal disorder. It is well documented that even moderately small displacements of the relative position of adjacent molecules can have a large effect on the transfer integrals [82]. The influence of lattice modes on intermolecular interactions, often referred to as non-local electron-phonon coupling, has been recognized by several authors [70,83,84]. In addition, it was revealed by dynamics simulations that the time-averaged value of transfer integrals is often of the same order of magnitude as their fluctuations [85,86]: as a consequence, even in crystalline materials the periodicity of the system at any given time is lost and the net result is a localization of the carrier wavefunction. Being the localization mainly due to low-energy lattice-vibration modulations of the transfer integrals, it is “transient”, in the sense that its lifetime *τ_l_* is basically determined by the frequency of the vibrations ω (*τ_l_* = 1/ϖ). This transient localization approach [14,15,87,88,89] pictures the carrier as exhibiting a more extended character on time scales longer than *τ_l_* but being localized by thermal disorder at shorter time scales, thus bridging between contrasting experimental evidence pointing towards the localized or the extended nature of the transport. Within the relaxation time approximation, charge mobility can then be expressed as:

(8) $$\mu =\;\frac{e}{k_{B}\cdot T}\cdot \frac{L^{2}}{2\cdot \tau_{l}}$$

where *L* is the localization length. Within this frame, mobility decreases with temperature but without assuming delocalized transport. This approach may also be applicable, as an alternative to hopping transport, to relatively disordered molecular systems which nonetheless exhibit a high mobility, where the assumptions behind hopping theories may not hold.

### Charge Transport in Columnar Phases

The description of the previous section, regarding charge transport in molecular materials, is only a very general outline focusing mostly on limiting cases. In fact, there are many models and computational approaches, based on first principles or phenomenological heuristics, that have not been mentioned but have been used: this very fact bears witness to the statement that the nature of charge mobility, in materials where molecular entities can be identified, is still a subject of debate. Therefore, the choice of the frame for the description of transport is usually dependent on the particular molecular systems, on the phases formed, on external conditions such as temperature range, on packing, and on degree of static and dynamic disorder. In addition, the experimentally measured value of mobility may rule out the use of some theoretical description. For example, the existence of Bloch-like electrons implies a mean free path between collisions which is much larger than the lattice spacing, i.e., the distance between neighboring molecules. In turn, this requires, besides a minimal presence of defects, also a low probability of thermal collisions, a condition valid at low temperature. Around room temperatures, the mean free path decreases and the requirement that it should be larger than the lattice constant poses a lower limit to the mobility for the applicability of the band model [12]. Considering typical parameters for molecular materials, this limit is μ_B_ ≳ 0.1^−1^ cm^2^·V^−1^·s^−1^. Hopping models rest on assumptions as well. One of them is that an energy barrier between sites exists. However, for t = λ/2 this barrier has already vanished and, considering zero-point energies, the barrier might disappear for even smaller electronic interactions [10]. Moreover, hopping implicitly assumes that electron transfer is slow enough for vibrational relaxation to be effective between consecutive charge transfer events. Considering the typical values for vibrational relaxation times and for the distance between molecules, this poses a higher limit [90] to the mobility if the hopping model is to be valid μ_H_ ≲ 0.1–1 cm^2^·V^−1^·s^−1^. The two limits clearly overlap in the range μ~0.1–1 cm^2^·V^−1^·s^−1^. Phases formed by disk-shaped molecules usually show a relatively high disorder, of different kinds and on different time and length-scales. In most cases, the measured mobilities are below 0.1 cm^2^·V^−1^·s^−1^, although an increasing number of charge mobilities that are one or two orders of magnitude higher than this value have recently been reported. For such reasons, mobility in discotic phases is usually modelled within the frame of the hopping model.

Given the relatively high disorder, hopping models must take into account the existence of charge traps [91,92]. Traps are sites where the drifting charges are localized for a certain time, thus affecting the observed current. Their origin may be intrinsic or due to chemical doping. Intrinsic, or structural, traps derive from defects in the ordered structure of the phase and include conformational variations, localized defects and grain boundaries. The traps of extrinsic, or chemical, origin include those due to intentional doping, often adopted in order to increase charge density, or to unintentional doping, such as those deriving from synthetic byproducts or from reactions with oxygen and water. The effect of traps on charge drift greatly depends on their density and depth, i.e., the energy stabilization provided by the trap with respect to the energy of the effective transport level. In general, shallow traps, i.e., those with a depth of a few *k_B_·T*, slow down charge transport while deeper traps contribute to the formation of a space-charge field, serve as recombination centers, but have a limited effect on steady state mobility because they fill easily and show detrapping on very long time scales.

As mentioned in the introduction, the use of mesophases as semiconductors represents an attempt at reaching a trade-off between the high molecular order required for optimal charge transport and the necessity to minimize grain boundaries, i.e., to extend the size of uniformly ordered domains towards the sizes necessary for their application in devices. This endeavor rests on the introduction of two features: a certain degree of flexibility in the molecular structure, in particular in the peripheral units, and a nano-segregation between the peripheral units and the cores. In such a way, the π-systems of the cores are not only in direct “contact” with each other, without the interposition of other order-disturbing molecular fragments, but in addition they may show, like in columnar phases, a relative orientation favouring intermolecular orbital interactions, thus increasing transfer integrals. At the same time, the conformational degrees of freedom within the intercolumnar nanophase introduce an element of mediation of the intermolecular interactions that, also during phase formation from the isotropic melt or from solvent evaporation, facilitates the development of larger ordered domains and the healing of structural defects. Charge transport in columnar phases is intimately connected to the different molecular structural features that determine the interplay of order and dynamics between the two nano-segregated environments. One additional advantage of columnar phases is that many materials often show a rich polymorphism. Larger uniformly oriented domains can then be obtained in the high-temperature, low-viscosity phases, and the macroscopic orientation in many cases can be frozen and transferred to the lower temperature phases by slow cooling. The net result is a material with good uniformity of orientation and high local order, both ingredients for a higher charge mobility.

The sensitivity of charge mobility in columnar phases to macroscopic order can also be understood considering early experimental work that recognized its highly anisotropic character [93,94]. Mobility along the column axes is roughly 2–3 orders of magnitude higher than in directions normal to the same axes. This is related to the preferential channels for charge flow provided by the intermolecular interactions of the π-systems within the columns, compared to the more “insulating” character of the intercolumnar environment. For this reason, columnar phases of organic molecules are often referred to as 1-D semiconductors in the literature. Although this may be desirable for some applications, it poses the problem that, when columnar order is disrupted by defects, charges become trapped, not being able to find alternative paths.

However, not only macroscopic order but also the degree of local order is important for good charge transport. Intracolumnar molecular distance, rotational disorder and extent of lateral displacement between adjacent molecules (disks) in the same column are different aspect of local order which were investigated [72]: they directly impact transfer integrals and are ultimately dependent on molecular structure. One important structural parameter is the length and branching of the peripheral chains. Down to a limiting point, beyond which crystalline phases are often formed, shorter and less branched chains favour inter and intracolumnar order, with a marked increase of charge mobility [95,96,97,98]. Also, the size of the central molecular cores has been recognized as an important parameter. In general, higher mobilities are observed for larger cores [99] and this is a consequence of several factors. The most obvious one is that extended π−systems provide a larger π-overlap, although caution should be exercised when considering this issue because transfer integrals do not depend on the degree of spatial overlap between molecules but instead on the degree of wavefunction overlap [100]. It is more realistic, then, to attribute the effect to a lower reorganization energy of large and rigid cores [78] and to the lower density of intracolumnar structural defects and shorter than average stacking distances, observed in discotic phases of larger core mesogens [101].

Intracolumnar order is not just related to the stacking distance but also to the relative molecular orientation. One of the parameters describing this kind of order refers to the rotational freedom of molecules around the columnar axis. Transfer integrals express the degree of wavefunction overlap between adjacent molecules and thus they depend markedly on the symmetry of the cores and on the relative rotation of neighbouring molecules. A complete rotational freedom then implies an intermolecular electronic coupling modulated in the time-domain by the rotational motions of molecules. In condensed phases, the extent and the dynamics of these rotational motions depend on the details of intermolecular interactions and of course on the temperature. If relative orientations corresponding to higher values of transfer integrals can be stabilized, charge mobility will be enhanced. This can be accomplished by introducing in the molecular architectures features that “lock” the azimuthal angles at the desired values via specific intermolecular interactions. In one example of this strategy [102], peripheral units of different polarity, alkyl and glycol chains, were alternated around cores with C_3_ symmetry. Preferential interactions among chains of the same nature belonging to adjacent molecules in the stack, stabilized azimuthal angles corresponding to higher transfer integrals, with a resulting increase of charge mobility. Similarly, the introduction of a regular helicity in the stack [52] can be viewed as a strategy for improving charge transport. The same effect can be achieved by using intermolecular hydrogen bonding [103]. However, the effect on charge mobility is not necessarily linked to optimal rotational order only, since also the stacking distance tends to decrease in the more ordered helical phases. Similar effects can be achieved exploiting intermolecular interactions due to permanent dipole moments [104]. The linking group between cores and peripheral chains can have a strong influence on order and mobility as well. As an example, by replacing oxygen with sulfur in hexaalkoxy substituted triphenylenes, a highly ordered helical plastic phase is observed at low temperatures, with a charge mobility 1–2 orders of magnitude higher than in the high temperature Col_h_ phase [105]. This mobility increase was attributed not only to a slightly decreased stacking distance in the helical phase but mainly to an increase of the core-core positional correlation length, from ~20 Å in the Col_h_ phase to ~300 Å in the helical phase [106]. Another example of the importance of the linking group comes from columnar phases formed by triindoles [107]. In this case, the substitution of bulky phenyl linking units with simple alkyl units produced an increase of mobility of nearly two orders of magnitude, despite the loss of a regular stacking order in the columns. The use of alkyne linkers led to further improvement, with shorter stacking distances and an increase of two more orders of magnitude in mobility.

The temperature dependence of charge mobility is commonly taken as a signature of the mechanism of charge transport. Better transport at lower temperatures is interpreted as due to an increase of the mean free path of charges between scattering events due to electron–phonon interactions in the frame of band-like transport. In contrast, higher mobilities at higher temperatures indicate activated transport and hopping of localized charges. In general, charge mobility in discotic phases is nearly temperature independent [108]. In part, this is due to the often narrow temperature ranges of existence of columnar phases, which do not allow a clear trend to be established. However, even in the cases where a more evident dependence is observed, care should be taken in interpreting data as a hallmark of a certain transport mechanism. In fact, as illustrated above, mobility in columnar phases depends on the subtle interplay of different kinds of positional and orientational order and dynamics, whose effect on mobility may exhibit a contrasting dependence, so that the observed temperature dependence of mobility may reflect, more than the transport mechanism, the effect of temperature on the details of phase structure and dynamics.

Charge mobility in discotic mesophases was also the subject of a number of theoretical studies. The most recent ones will be described in Section 5. Here we will just mention how the effect of positional and orientational order on transfer integrals was studied within the frame of the Marcus hopping theory [4,72]. In addition, the complexity deriving from the simultaneous effects of static and dynamic disorder often requires the use of multiscale approaches combining the use of quantum chemistry calculations, molecular dynamics and Monte Carlo simulations [109,110,111]. Moreover, electron–phonon coupling in columnar phases is, in fact, usually strong but since nuclear dynamics is often on the same time-scale as electron dynamics allows neither a treatment that considers static disorder, nor the averaging of nuclear motion in electronic Hamiltonians and, as a consequence, may require more specific modelling approaches [112,113].

## 4. Measurement of Charge Mobility

No progress in any scientific field can be achieved without accurate and reliable measurements of observable quantities. Of course, this is true for the performance of organic semiconductors as well. Although from the simple definition of charge mobility one might expect straightforward measurements, this is not the case for several reasons. In fact, different measurement techniques probe charge mobility at different time- and length-scales, which in itself would be enough to expect contrasting results. In addition, almost all techniques for measuring charge mobility rest on assumptions that must be fulfilled in order to obtain meaningful data, and this is not always the case. Moreover, as if this would not be enough in itself, in many cases the collected data depend on the interaction of the investigated materials with the surfaces of electrodes, because of charge injection issues and/or because the morphology of the first few molecular layers at interfaces determines the measurement outcomes. In other words, especially, but not only, when the size of samples is particularly small, what is probed during measurements is the material/electrode interface more than, or as much as, the material bulk property. These interface properties are not only dependent on the material chosen as the electrode, but also on the details of sample preparation, a fact that certainly complicates the interpretation of results. For all these reasons, quite often the experimental technique used for quantifying charge mobility is explicitly specified and one talks about time of flight (TOF) mobility, organic field-effect transistor (OFET) mobility and so on. In the following, the most common methods used for measuring charge mobility in organic materials will be reviewed, underlying their respective advantages, assumptions and limitations.

### 4.1. Field-Effect Transistors

**Figure 8:**
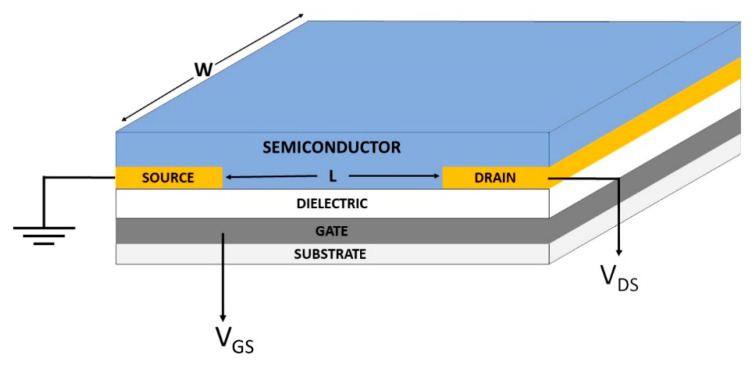
Schematic illustration of a field-effect transistor.

Due to its (apparent) simplicity, this technique has been used extensively for the assessment of charge mobility in organic materials [9,114,115,116,117,118]. In an OFET the organic semiconductor constitutes the conduction channel of a transistor. The working principles of the device will be briefly illustrated in reference to positive charges as charge transporters, but by changing the sign of the applied voltages the same considerations apply to electrons. Figure 8 is a schematic illustration of a device, although other architectures are possible, with different positioning of the electrodes above or below the semiconductor. The electrodes are usually metallic, often obtained by deposition of Au, but other metals or even conducting polymers have been used. The choice of the electrodes is extremely important because it determines the contact resistance, when the work function of the electrode is considered in combination with the frontier orbital energies of the semiconductor. The insulating layer can be made of metal oxides or polymers. By applying a negative voltage on the gate electrode (with respect to the source, *V_GS_*) positive charges will accumulate at the dielectric/semiconductor interface. Their number, besides that on the magnitude of *V_GS_*, will depend on the dielectric constant and the thickness of the dielectric. In these conditions, when a voltage bias *V_DS_* is applied between the drain and the source electrodes, a current *I_D_* should be observed. However, for small values of *V_DS_* there is almost no current because charges get pinned at traps and defects within the channel. Only when *V_DS_* reaches a certain threshold value *V_th_*, the current *I_D_* starts increasing according to Ohm’s law. In this linear regime, the current is given by:

(9) $$I_{D}^{l}=\mu \cdot C\cdot \frac{W}{L}\;\cdot \left[V_{DS}\cdot \left(V_{GS}-\;V_{th}\right)\right]$$

where *C* is the capacitance of the dielectric layer, μ is the charge mobility and W and *L* are the geometric parameters of the channel, as shown in Figure 8. This is valid until the charge in the channel is approximately constant, i.e., for |*V_DS_*| << |*V_GS_* − *V_th_*|. In other words, the transistor operates as a resistor with a conductivity which increases by increasing the amount of mobile charges via *V_GS_*. However, when |*V_DS_*| > |*V_GS_* − *V_th_*| a so-called saturation regime is reached, when all charges generated in the channel via *V_GS_* are depleted and a constant *I_D_* independent from *V_DS_* is measured: the device can be thought of as a constant current generator, with the current determined by *V_GS_*. In this regime the current is given by:

(10) $$I_{D}^{s}=\mu \cdot C\cdot \frac{W}{2L}\;\cdot {\left(V_{GS}-\;V_{th}\right)}^{2}$$

**Figure 9:**
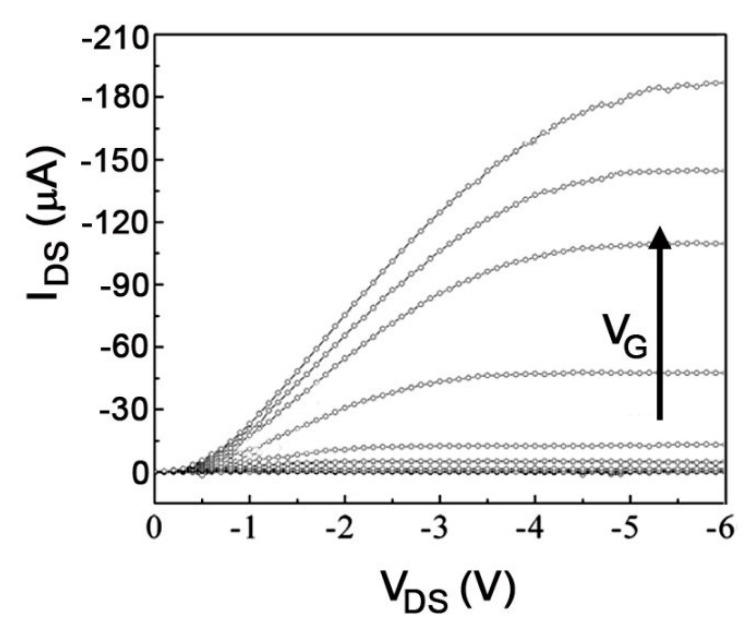
Typical variation of *I_DS_* as function of *V_DS_* at different values of *V_G_* in a field-effect transistor.

Typical characteristic curves showing the variation of the drain current as a function of *V_DS_* for different values of *V_GS_* are shown in Figure 9, where both regimes can be observed. Equations (9) and (10) can be used to extract charge mobility. They are valid provided that the electric field due to *V_DS_* is much smaller than the gate field, a condition usually achieved by choosing appropriate geometric parameters for the channel. In addition, a charge mobility independent from charge density is assumed. However, the fulfillment of these conditions is far from enough in order to obtain reliable measurements of charge mobilities and care should be exerted in treating experimental data [119,120,121,122]. Among other factors, device fabrication should be optimized, in order to minimize threshold voltages and contact resistances

In OFETs, charge transport occurs in the first few molecular layers of the semiconductor, at the interface with the gate dielectric, and from this point of view this measurement technique can be considered as working in 2-D. In anisotropic materials results are heavily dependent on the degree of macroscopic order and orientation of crystal axes at such an interface. With the aim of controlling this feature, several deposition techniques were developed, including the use of monolayers of a third substance between dielectric and semiconductor. The measurements of drift mobilities are performed over length-scales of the order of 10–100 μm (channel length) and time-scales of the order of the second. The typical charge densities obtained in OFETs, of the order of 10^19^–10^21^ cm^−3^, are higher than in many other techniques and this makes the measurements somewhat less sensitive to the effect of traps, at least to those due to local disorder, because with these higher carrier concentrations residual tail states in the density of energetically available states are more easily filled up.

### 4.2. Time of Flight

**Figure 10:**
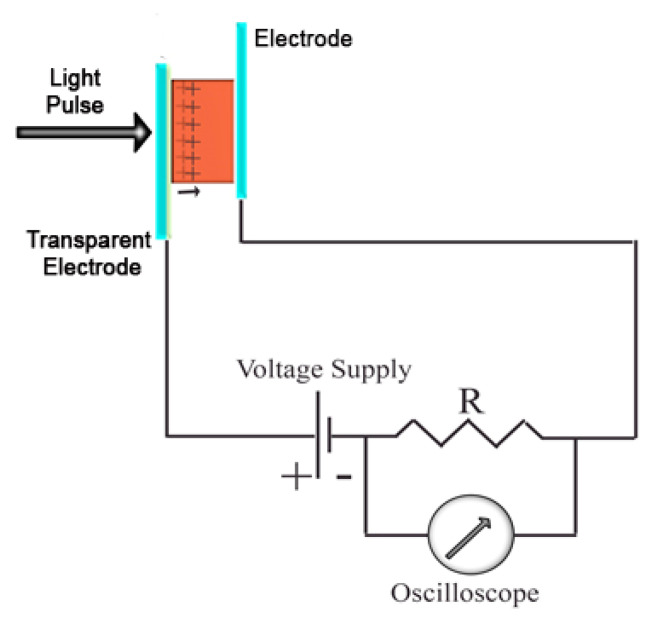
Experimental setup for time-of-flight measurements.

**Figure 11:**
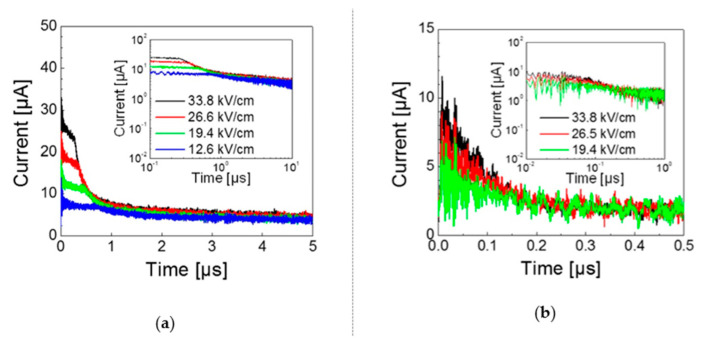
Typical transient photocurrent measured in time-of-flight (TOF) experiment on 1,4,8,11,15,18,22,25-octahexyltetrabenzotriazaporphyrin in the case of (**a**) non-dispersive transport, (**b**) dispersive transport. Insets show double logarithmic plots. (Adapted from reference [125] with permission from Elsevier. Copyright 2017 by Elsevier).

One of the most widely used techniques for measuring charge mobility in organic semiconductors is the so-called time-of-flight (TOF) method. In this technique, the semiconductor is sandwiched between two electrodes, at least one of which is transparent or semitransparent, as illustrated in Figure 10. When a short light pulse impinges on the semiconductor through the transparent electrode, a certain number of charges of opposite signs are photogenerated. It is assumed that the extinction coefficient of the semiconductor is high enough, or its thickness d large enough, that the light penetration depth is much smaller than d. In these conditions, if a DC potential difference V is applied on the two electrodes, charges of the sign opposite to the sign of the potential of the electrode exposed to the light pulse, are quickly depleted through such electrode. Charges of the other sign travel the whole thickness of the film, generating a current that can be detected through a resistor in series with the sample. In an ideal case of non-dispersive transport, a transient photocurrent like the one shown in Figure 11a is recorded, from which the transit time *t_E_* of the charges through the semiconductor can be easily extracted. In not uniformly oriented samples of anisotropic materials, or if traps are present, photogenerated charges will show different transit times and the shape of the transient photocurrent will look more like the one shown in Figure 11b. In this case, the transit time may still be extracted if the data are plotted in double logarithmic graphs [123], as shown in the inset of Figure 11b. Once the transit time is measured, mobility can be obtained from its very definition as:

(11) $$\mu =\;\frac{d^{2}}{V\cdot t_{E}}$$

Besides the fact that the light penetration depth must be much shorter than *d*, other conditions are required for obtaining meaningful and informative data. For obvious reasons, the transit time *t_E_* must be shorter than the charge lifetime and longer than the RC constant of the circuit. In addition, the photogenerated charge density must be small enough to not alter significantly the electric field due to the potential difference *V*, otherwise distorted current transients may be recorded and the transit time not correctly extracted. This is usually accomplished by limiting the light energy, so that the photogenerated charge (that can be obtained by integrating the TOF transient) can be much smaller than the charge due to the geometric capacitance of the measurement cell [124].

The TOF method has the advantage of not requiring injecting contacts, whose effectiveness is often uncertain. Moreover, it can easily be used to measure mobilities of both holes and electrons within the same sample. Often, transit times are recorded as a function of the applied voltage, but this is done to check that Equation (11) is obeyed more than to extract a field dependence of mobility, also because the range of the used applied voltages rarely spans more than one order of magnitude and short transit times are hard to measure experimentally [126]. In addition, as shown in Figure 11a, at higher applied voltages the number of photogenerated charges increases and the signal/noise ratio improves. This is due to a field-dependent photogeneration efficiency. In fact, there is wide experimental evidence that in organic semiconductors the absorption of light leads to the formation of excitons, quasiparticles consisting of electron/hole bound pairs, within a subpicosecond excited state dynamics. Excitons can diffuse and they can recombine to the ground state or dissociate, leading to electrons and holes that can contribute to charge transport, if the two charges can escape the mutual Coulomb attraction. Hence, dissociation is favoured by an electric field, pushing the opposite charges in different directions, and it can occur via different mechanisms, including exciton–exciton interaction in the bulk or charge separation/transfer at interfaces with electrodes.

Given the requirements listed above, the sample thickness is usually between 1 and 20–30 μm, so that TOF probes macroscopic 3-D drift mobility in the time scale of 10^−7^–10^−4^ s, at low charge densities around 10^13^–10^15^ cm^−3^. This method has been used extensively to measure charge mobility both in smectic [25,127] and in columnar [26,125,128,129] mesophases.

### 4.3. Space Charge Limited Current (SCLC)

When charges are injected in a dielectric from an electrode, under the effect of an electric field they may drift away towards the opposite electrode. However, if the injecting electrode is efficient, the injection rate of charges can be higher than their depletion rate due to the field, leading to an accumulation of charge in the dielectric region next to the injecting electrode. This space charge will limit the injection and the current, whose value will be basically determined by the charge mobility in the dielectric [74]. It should be stressed that this assumes that charges are more easily injected from the electrode into the semiconductor than they can be carried away from the current in the device, a condition usually indicated by saying that the contact is ohmic. In other terms, the current must be controlled by the mobility of charges in the semiconductor and not by the ease of injection at the electrode/semiconductor interface. When this condition is fulfilled, neglecting the effect of charge traps, the Mott-Gurney law [130] will describe the dependence of the measured current density J on the applied voltage:

(12) $$J=\;\frac{9}{8}\mu \epsilon \frac{V^{2}}{d^{3}}$$

where *ε* is the permittivity of the semiconductor. Often, a field dependence of the mobility is observed. If it can be attributed to the presence of disorder with shallow traps only, i.e., traps with an energetic distribution not much larger than *k_B_·T*, the field dependence of mobility can be expressed as [131]:

(13) $$\mu =\;\mu_{0}\exp\left(\gamma \;\sqrt{E}\right)$$

where *μ*_0_ is the mobility in the limit of vanishing electric filed *E* and *γ* is a parameter expressing the strength of the field dependence. Equation (13) is often inserted in Equation (12) for data evaluation. However, a field-dependent mobility may also hide the effects of deep traps [132] or charge density [133], and in these cases more detailed models are needed for a description of mobility, combined with a wider experimental evaluation in the range of the parameters [134,135,136].

**Figure 12:**
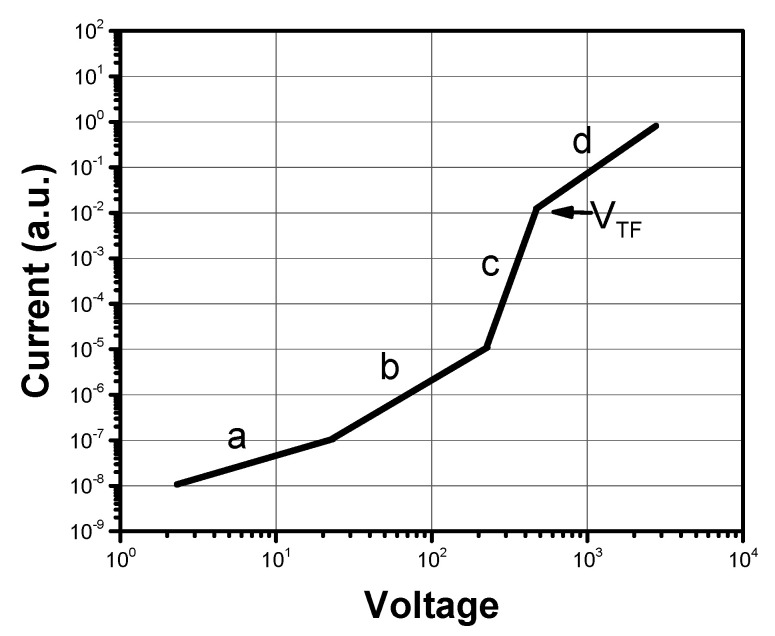
Schematic characteristic curve in a space charge limited current (SCLC) experiment in which the different regime of variation of the current as function of the applied voltage are shown: (**a**) ohmic, (**b**) trap-limited SCLC, (**c**) trap-filling, (**d**) trap-free SCLC.

Organic semiconductors are often disordered to some extent and contain impurities. The effect of traps can be evident in experimental characteristic curves, as shown schematically in Figure 12. In general, for low applied voltages, a current varying linearly with the applied voltage, according to Ohm’s law, should be observed. The absence of a clear ohmic regime at low applied voltages may be a signal of an injection-limited current. Upon increasing V, a first space charge limited current (SCLC) regime may be entered, where the current is limited by the presence of traps. The current in this regime is affected by the trapping-detrapping process and depends on the number and the energetic distribution of traps. For a further increase of applied voltage, the density of charges may reach values high enough to gradually fill the traps. During this trap-filling regime the J(V) dependence may become even steeper than what is predicted from Equation (12), until finally, for voltages higher than V_TF_, when all traps have been filled and do not contribute to transport, a second SCLC regime is observed where a mobility μ can be measured [137,138].

Given the assumptions and the discussion developed above, a certain number of precautions should be taken in order to avoid erroneous results. Injection limited currents may show a voltage dependence similar to the one observed in SCLC, hence the requirement of an ohmic injecting contact is of paramount importance. Efficient injection is usually achieved upon aligning the transport level (HOMO or LUMO) of the semiconductor with the work function of the electrode, although this might not be sufficient, since the orientation of molecules at the interface might play a very important role in determining the injection efficiency. Ohmic contact are particularly relevant in thinner samples, where higher currents are necessary to reach the SCLC regime. Devices must also be unipolar, because otherwise the current will contain contributions from both holes and electrons. This is accomplished by using a non-injecting contact for the unwanted charges. In thin devices and when the mobility (current) is high, in particular, the series resistance of the measuring circuit should be minimized, including the resistance of the electrode itself. A built-in voltage may exist in devices, often due to a mismatch of the work functions of the two electrodes. In this case, the built-in voltage should be subtracted from the applied voltage when analyzing data. Again, this problem is particularly relevant for thin samples, where lower voltages are used. Finally, Equation (12) predicts an inverse dependence of the current on the cube of the sample thickness. It is always advisable, when possible, to check this dependence on samples of different thickness at the same applied field, to increase the reliability of the measurement against effects due to injection issues. Recently, the thickness dependence of the SCLC currents has also been related to the degree of spatial disorder in the semiconductor [139]. The SCLC technique probes 3-D drift mobility on a length-scale between 10^2^ and 10^4^ nm, with charge densities around 10^15^–10^16^ cm^−3^ and on time scales of the second. It can separately measure the charge mobility of holes and electrons in the same material and in thin as well as in thicker samples. However, despite its apparent simplicity, its application requires careful implementation and precautions if reliable results are to be obtained [140].

**Figure 13:**
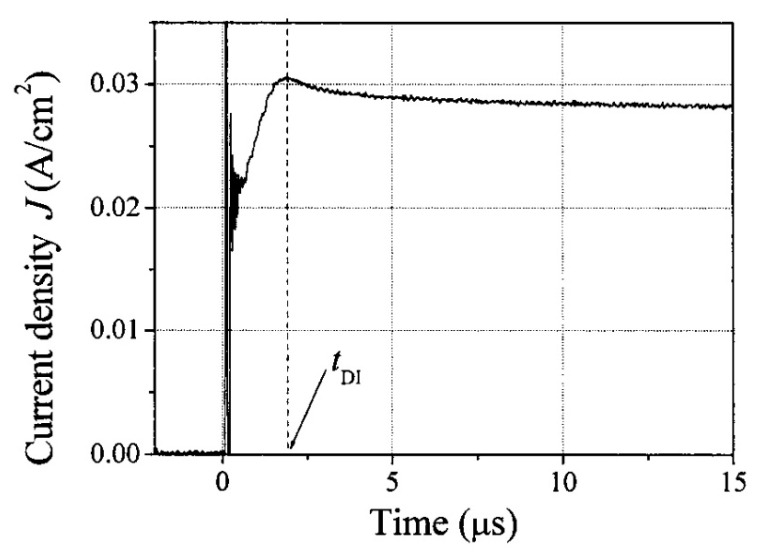
Typical characteristic curve of a dark injection space charge limited current (DI-SCLC) measurement. (Reprinted from reference [143] with permission of AIP Publishing. Copyright 2003 by AIP Publishing).

Charge mobility in the SCLC regime can also be obtained from transient, instead of steady-state, measurements. The method goes under the name of the dark injection space charge limited current (DI-SCLC) technique and calls for the application of a step-voltage to a sample with an ohmic contact. Right after the application of the pulse, the current increases because the charge packet initially injected experiences an increasing field due to its own space charge. After reaching a maximum at a time t_M_, in the absence of traps the current gradually relaxes towards a constant value, as shown in Figure 13. It can be shown [141] that the transit time *t_E_* of a carrier through the thickness of the device is related to t_M_ according to t_M_ = 0.786·*t_E_*, so that Equation (11) can then be used to extract the mobility [142,143,144].

### 4.4. Charge Extraction by Linearly Increasing Voltage

**Figure 14:**
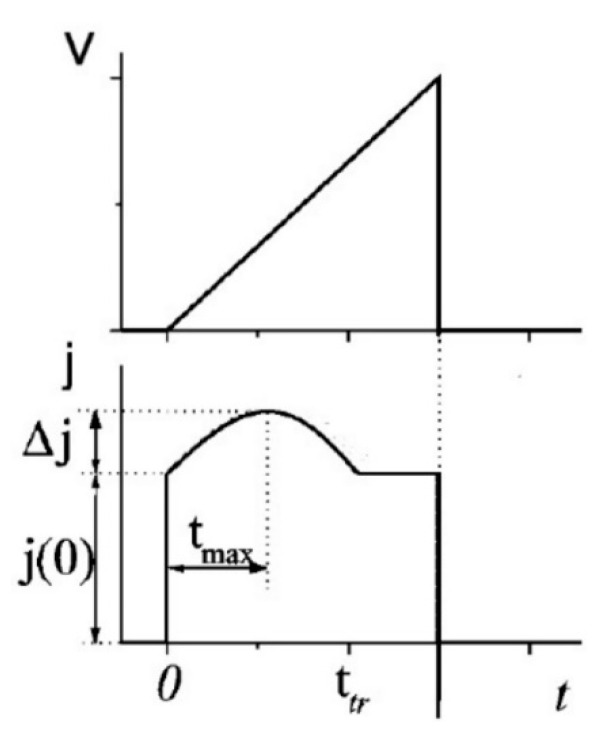
Scheme of the applied triangular pulse and measured current in CELIV experiments. (Adapted with permission from reference [145]. Copyright 2000 by American Physical Society.).

When a train of triangular, linearly increasing voltage pulses of slope A = dV/dt is applied to a semiconductor with blocking contacts, the first pulse charges the electrodes. Any of the following pulses causes a current due to the extraction of equilibrium charges, as schematically shown in Figure 14. The initial current step for which the current jumps to the value *J*_0_ is the usual response tied to the geometric capacitance. The current then increases reaching a maximum *J = J*_0_ *+* Δ*J* before decreasing to a stable plateau after the extraction of charges. The time *t_max_*–*t*_0_ for which the current reaches a maximum can be used to estimate the mobility [145,146]. For different limiting regimes of high (Δ*J* >> *J*_0_) and low (Δ*J* << *J*_0_*)* conductivity the relationship between charge mobility and t_max_ can be shown to be different [147]. However, in most experimental studies Δ*J*~*J*_0_ and from numerical estimations one can derive:

(14) $$\mu =\;\frac{2\cdot d^{2}}{3\cdot A\cdot t_{max}^{2}\;\left(1+0.36\cdot \frac{\Delta J}{J_{0}}\right)}$$

where *d* is the sample thickness. This method requires a sufficient density of intrinsic carriers, a condition not often encountered in organic semiconductors. For this reason, in some cases the voltage pulse is synchronized with a charge-photogenerating light pulse, in a technique known as photo-CELIV [148].

Given the fact that equilibrium charge carriers are probed, CELIV is less affected by dispersive transport issues [149]. In addition, transparent electrodes are not necessary but in some cases it may be difficult to distinguish hole mobility from electron mobility in ambipolar semiconductors. Although several experimental parameters can be varied, there are practical limits for the mobilities that can be measured with this method. For low mobilities, the peak maximum should still be observed at times shorter than the pulse length and for high mobilities the extraction time should still be longer than the RC constant of the circuit.

### 4.5. Time-Resolved Microwave Conductivity

In the absence of charges free to move, microwaves propagate through matter with almost no attenuation. However, when free charges are present, they drift under the effect, and at the frequency, of the microwave field. This dissipates energy and the attenuation of the power of the microwave beam is directly proportional to both the volume density and the mobility of the charges. If the density of charges is known, it is possible to evaluate their mobility by synchronizing the measurement with an event that generates the charge transporters. In the most common application of this technique [80,150,151,152,153], free charges are generated by an electron beam pulse of ~10 nanoseconds and the method is referred to as pulse-radiolysis time-resolved microwave conductivity (PR-TRMC). In this case, the number of generated charges can be evaluated from the radiation dose, also with the use of correction factors that include an estimation of the recombination during the electron pulse. Mobility can then be extracted from microwave attenuation data. This method has the great advantage of not requiring electrodes. However, the contribution of holes and electrons to the detected signal cannot always be clearly separated and the resulting mobility contains contributions from both types of charges.

The typical microwave frequency used are in the 10–50 GHz range. This means that, even for relatively high mobilities, under the effect of the AC field charges drift within a distance of the order of 1 nm. As a consequence, the mobility derived from TRMC is almost unaffected by macroscopic disorder and it is often considered as un upper limit of mobility at low fields when compared to mobilities measured in the same material via other techniques that probe transport over much longer distances and where defects and grain boundaries can play a dominant role. In a different version of the technique [154,155] charges are generated by a light pulse, although in this case it is more difficult to determine the density of photogenerated charges in order to extract mobility.

### 4.6. Admittance Spectroscopy

In this technique [156], a voltage is applied on a sample placed between two electrodes. Often, one electrode is injecting (ohmic contact) for one type of charge, let’s say holes, while the other one does not inject charges of the opposite sign, and to a first approximation the device current can be attributed to charges of the same sign only. The applied voltage contains a small amplitude (10–100 mV) AC component at a frequency f = ω/2π superimposed to a usually more intense DC bias. The current response of the device is then monitored as a function of the bias and of the frequency of the AC voltage. In general, the current will contain two components, one in phase (conductance) and another one in quadrature (susceptance) with the applied voltage. Both signals may contain the signature of charge mobility. Let us consider the susceptance B = ϖ·C first, where C is the capacitance. In fact, the AC voltage probes the space charge in the sample, due to both the AC and the DC voltages, on a time scale that should be compared with the transit time *t_E_*. For frequencies ϖ < 1/t_R_, where t_R_ is an admittance relaxation time close to *t_E_*, the detected current contains the effect of the AC voltage on the space charge. Such a contribution to the current lags behind the AC voltage, so that it lowers the capacitance, as for inductive contributions to the susceptance. For higher frequencies ω > 1/t_R_ this inductive-like contribution to the current disappears and the capacitance increases to a stable value, corresponding to the geometric capacitance. In conjunction with appropriate modelling [157,158] which relates t_R_ with *t_E_*, the frequency corresponding to the step in capacitance was related to the mobility of charges in organic materials. While the capacitance shows an increasing step with increasing frequency, conductance shows a decreasing step at the same frequency, due to the loss of the contribution of the charges injected by the AC voltage. This decreasing step might be partially masked by the increase of conductance with increasing frequency when the behavior is dominated by the series resistance of the device. Further studies [159,160] highlighted the role of admittance spectroscopy in probing the details of charge transport in organics, in particular the influence of charge traps.

In order to use this technique effectively to measure charge mobility, the inductive-like contribution to capacitance must be large, which implies a good ohmic contact at the injecting electrode. In addition, the maximum available frequency of the impedance analyzer used limits the measurements to samples of a certain minimum thickness, which increases with increasing mobility. Moreover, the deep trap concentration should be low enough to ensure the observation of a clear capacitance step and the frequency above which the effect of traps is negligible should be much lower than 1/t_R_.

## 5. Recent Advances in the Study of Discotic Liquid Crystals

In the present section, advances and innovations emerged in recent years in the field of discotic liquid crystals (DLC) will be presented, focusing mainly on the properties that make DLC suitable for use in actual devices.

### 5.1. Mesophase Order

Positional/orientational order is one of the most studied properties of DLC because it is strictly related to charge transport and therefore to the possibility of using DLC in devices. Mesophase order depends on several different factors and in this section, the most recent research developments in this area will be discussed.

The interactions among the side chains of the discotic molecules play an important role in improving or worsening the intracolumnar order because the relative position and orientation between two successive discotic molecules will be determined, among other factors, by the interaction energy between side chains. In other words, one molecule will rotate with respect to the next one in the column to achieve a lower energy, and the relative positions of the side chains is a key factor in defining this aspect of molecular organization. This will change the overlap between the orbitals of two adjacent molecules and therefore charge transport properties. An example of this dependence was given by Zhang and coworkers, who studied compounds containing the triphenylenetris(naphthalene imidazole) core, yielding n-type semiconductor DLC [161,162]. They replaced the branched chains with linear ones, measuring a mobility of 1.3 × 10^−3^ cm^2^·V^−1^·s^−1^ [162], more than one order of magnitude higher than the one reported for compounds bearing branched chains, confirming that also lateral chains can influence the properties of mesophases. In the same paper, they established through GIWAXS studies that two adjacent molecules in a column are rotated by an angle of 13° respect to each other. In addition, density functional theory (DFT) methods were used to calculate reorganization energies and transfer integrals versus rotation angle, showing that the rotation angle of 13° measured by GIWAXS corresponds to high values of transfer integral and to low values of total energy of the dimers, providing an explanation for the high charge mobility measured in mesophases formed by this class of molecules.

**Figure 15:**
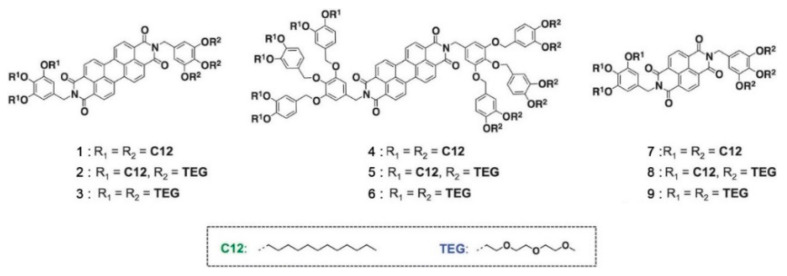
Perylenediimide and naphthalenediimide cores with hydrophobic dodecyl (C12) and hydrophilic triethyleneglycol (TEG) chains (Adapted with permission from reference [163], published by Royal Society of Chemistry. Copyright 2016 by Royal Society of Chemistry).

**Figure 16:**
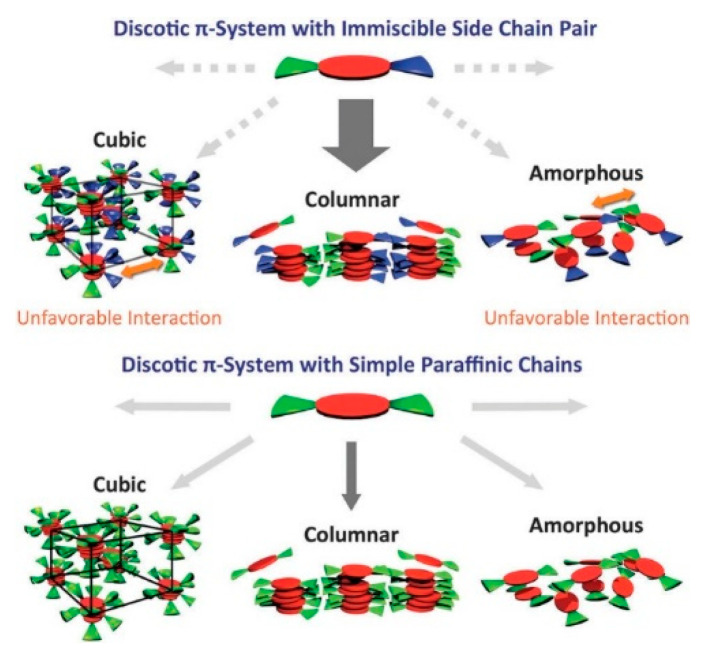
Proposed model to explain the mesophase formation in Perylenediimide or naphthalenediimide with hydrophobic hydrophilic chains (Adapted with permission from reference [163], published by Royal Society of Chemistry. Copyright 2016 by Royal Society of Chemistry).

Another example of how the mesophase order can be affected by the nature of the side chains was given by Sakurai and coworkers [163], who studied the mesomorphic properties of a series of compounds formed by perylenediimide or naphthalenediimide cores with hydrophobic dodecyl (C12) and hydrophilic triethyleneglycol (TEG) side chains (Figure 15). They observed that when the ratio between side chain and core volumes is smaller, columnar mesophases are formed for any assortment of side chains, while when the ratio of side chain to core volumes is higher, columnar mesophases are formed only if the hydrophobic and hydrophilic side chains are both present in the molecules. In fact, the derivatives that bear only one kind of side chain form micellar cubic, crystalline, or isotropic liquid phases because they are thermodynamically more stable than columnar phases. When both hydrophilic and hydrophobic chains are present in the same derivative, the formation of columnar mesophases is thermodynamically favored because in these phases the interaction between the immiscible hydrophilic and hydrophobic chains can be minimized (Figure 16).

**Figure 17:**
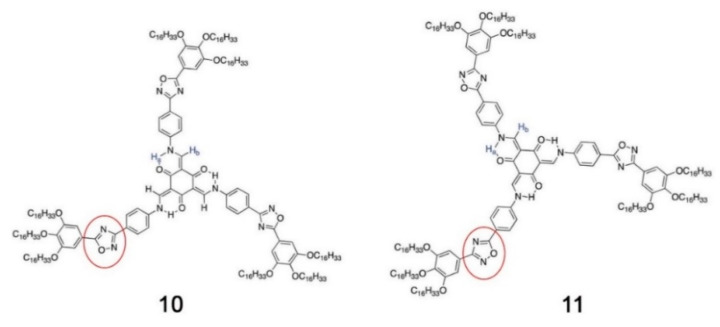
Molecular structures of the star-shaped oxadiazole-based tris(N-salicylideneaniline)s based Isomers (Adapted with permission from reference [164] published by Royal Society of Chemistry on the behalf of the Centre National de la Recherche Scientifique (CNRS) and the RSC. Copyright 2017 by Royal Society of Chemistry).

Even minor variations in the chemical structure, as in different isomers or with side chains linked in different positions, can induce pronounced effects in the mesomorphic properties of compounds [164,165,166]. For example, Pathak and coworkers studied couples of star-shaped oxadiazole-based tris(N-salicylideneaniline)s that differ only for substitution on the 3,5-positions of the central 1,2,4-oxadiazole, as highlighted in Figure 17. Because of the difference in the resulting electronic distribution in the two isomers, compound **10** forms a columnar hexagonal phase, while **11** forms a columnar rectangular one [164].

**Figure 18:**
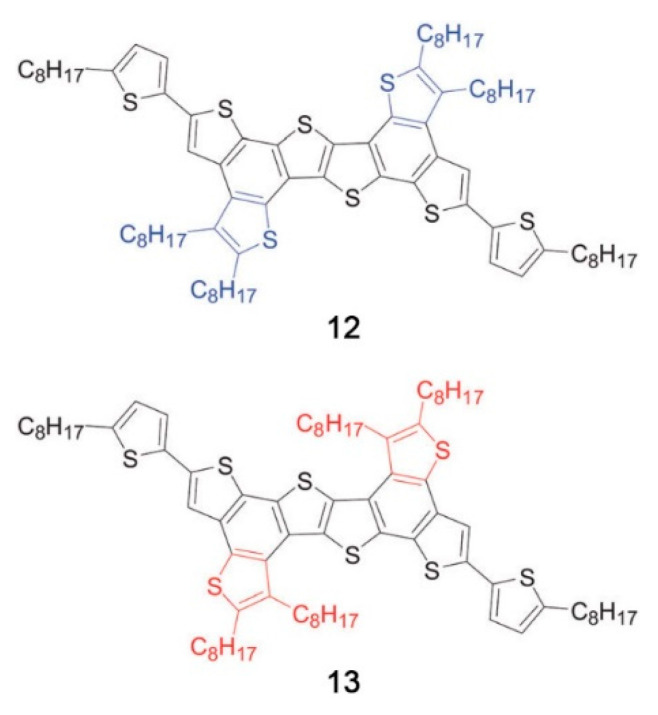
Fused-thiophene Isomers with different position of the fused thiophene rings. (Adapted from reference [166] published by the PCCP Owner Societies).

Dang and coworkers, instead, analyzed two isomers containing fused-thiophene cores (Figure 18), that differ only for the relative position of two of the fused thiophene rings. This change induced two completely different core geometries, planar in the case of **12** and distorted in the case of **13**, and consequently also the resulting mesophases are different: the strong π–π interaction among the planar cores of **12** allows the formation of a well-ordered columnar mesophase, while in **13** the repulsion among sulfur atoms reduces the π–π interaction between the cores, inducing the formation of a more disordered columnar mesophase. The more planar conformation of **12** influences the luminescent properties in films, because its stronger π–π stacking quenches the emission, and therefore a higher quantum yield and a longer lifetime were observed for **13**. A difference was also observed in charge mobility measured in OFET, since **12** showed a mobility of about 4 × 10^−3^ cm^2^·V^−1^·s^−1^ while the mobility of TF2 was 8 × 10^−4^ cm^2^·V^−1^·s^−1^ [166].

**Figure 19:**
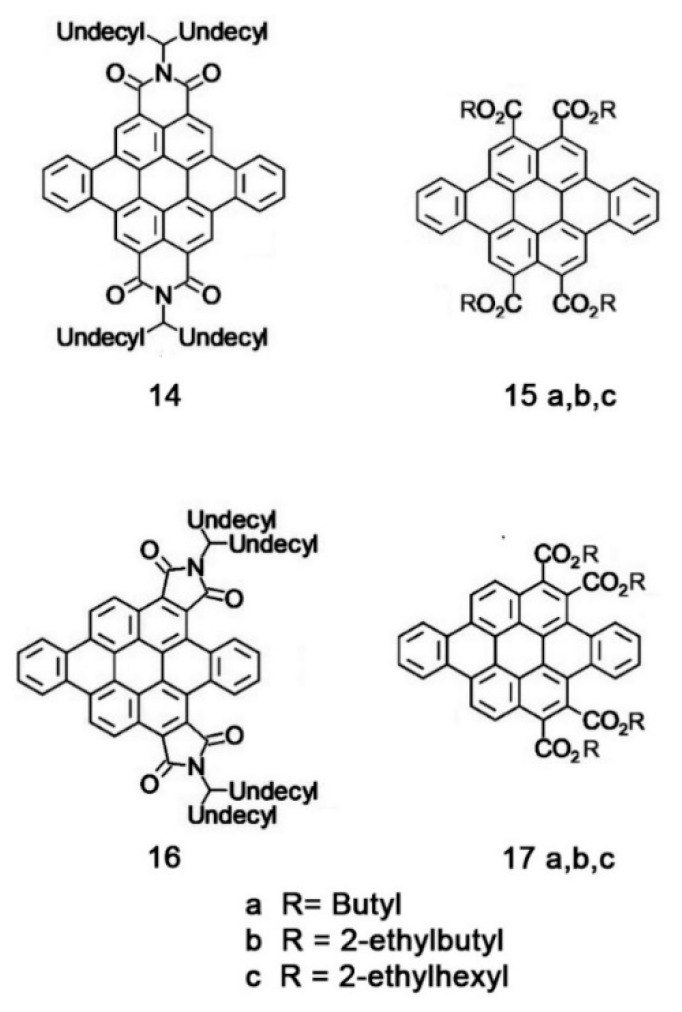
Symmetric and asymmetric dibenzocoronene-tetracarboxylic alkyl esters and imides (adapted from reference [165] with permission of John Wiley and Sons. Copyright 2018 by John Wiley and Sons).

**Figure 20:**
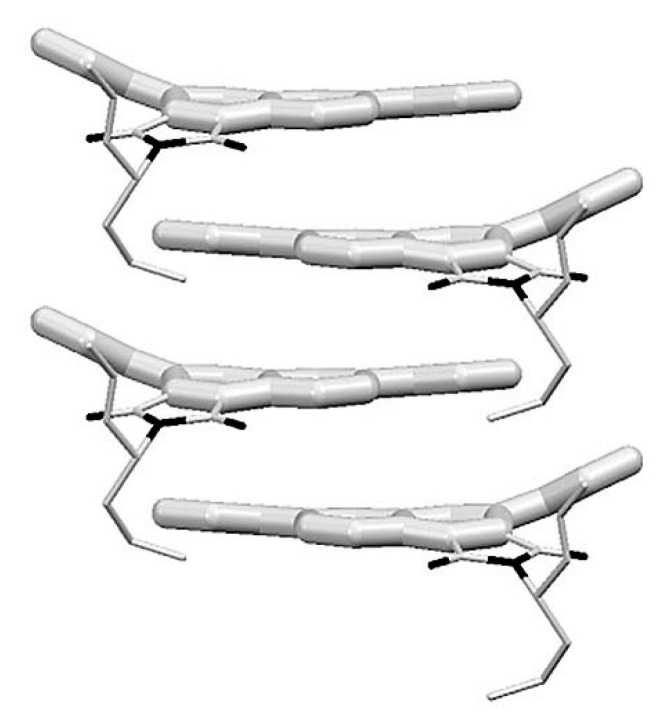
X-ray derived stacking for a compound homologue of **16**, with the same core but with shorter side chains in order to induce crystallization (adapted from reference [165] with permission of John Wiley and Sons. Copyright 2018 by John Wiley and Sons).

Along the same lines, Ferreira and coworkers studied the effect of the position of side chains in dibenzocoronene-tetracarboxylic alkyl esters and imides, comparing the mesomorphic properties of the symmetric compounds **14** and **15 a,b,c** with those of the asymmetric homologues **16** and **17 a,b,c** (Figure 19). From crystallographic observations, they deduced that the core of compounds **16** and **17**, is bent because of steric hindrance. The loss of planarity is usually detrimental for the stability of mesophases, that can become more disordered and stable in smaller temperature ranges. In this case, instead, something different has been observed, as the asymmetric alkyl esters **17 a,b,c** form hexagonal columnar phases within temperature ranges wider than those exhibited by the symmetric ones **15 a,b,c,** while compound **16** forms the columnar mesophase at lower temperature than its symmetric counterpart. The worsening of the mesogenic properties of asymmetric compounds is avoided because in the columnar structure molecules assume a slipped arrangement (Figure 20) and, therefore, the π–π interactions are preserved [165].

**Figure 21:**
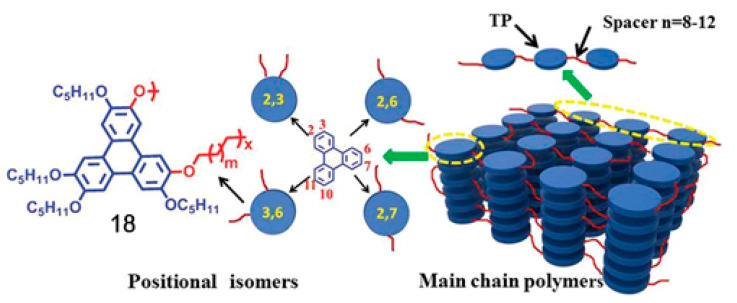
Molecular Structure of polyethers polymers carrying a dihydroxytetraalkoxytriphenylene fragment (adapted with permission from reference [167], published by Royal Society of Chemistry. Copyright 2016 by Royal Society of Chemistry).

In polymers, dyads and triads the mesophase order can be also affected, even heavily, by little modifications on the linkers that connect the different molecular fragments. Zhang and coworkers [167] studied the structure/properties relations in a series of main chain DLC polyethers, carrying a dihydroxytetraalkoxytriphenylene fragment, Figure 21. In particular, they studied how the position of the substitution and the spacer chain length can influence and modulate mesophase properties. They prepared different polymers with different combinations of substitution positions and spacer chain lengths, observing that the more ordered phases were obtained when the spacer length was twice as long as the alkoxy lateral chain of the dihydroxytetraalkoxytriphenylene fragment. A similar trend was observed also from Wang and coworkers, who studied the structure/properties relationship in different dimers of triphenylene derivatives [168,169]. They described the properties of a series of dimers synthetized by coupling two hexapropoxytriphenylene units (HAT3) by chains of different length. Similarly, they prepared a second series of dimers coupling two hexabutoxytriphenylene (HAT4) units and a third one linking two hexalkoxytriphenylene (HAT5) units [169]. They observed that when the length of the linking chain is exactly twice the length of the alkoxy side chain of the triphenylene units, the largest enthalpies of transition, the smallest intracolumnar spacings and the highest charge-carrier mobilities among the dimers of each series is obtained. These observations can be understood by considering that when the linking chain is twice as long as the alkoxy side chain, the dyad is quite similar to two monomers of the corresponding triphenylene unit, structural perturbations are minimized and more stable and ordered mesophases are obtained.

**Figure 22:**
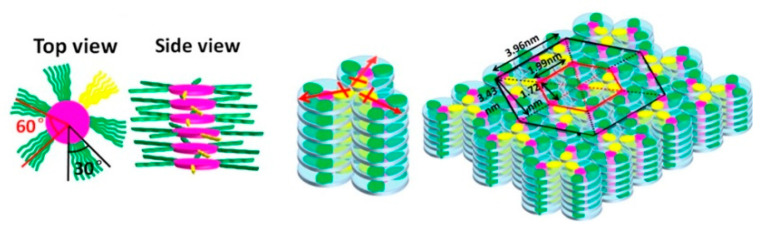
Proposed model to describe the superlattice formation in HAT 5 modified compounds (reprinted with permission from reference [171]. Copyright 2017 American Chemical Society).

Another parameter that strongly influences mesophase order is represented by the possible presence of an electric dipole [170,171,172]. Zhao and coworkers, for example, synthetized a series of fourteen different dibenzotetracenes with different substituents on lateral chains [170]. They observed that the mesophase formed by the compound in which two fluorine atoms were inserted as substituents, inducing a lateral dipole, showed a mobility of the order of 10^−4^ cm^2^·V^−1^·s^−1^, while in the mesophase formed by the analogue non-polar compound the mobility was of the order of 10^−2^ cm^2^·V^−1^·s^−1^. This was attributed to the fact that, in the polar compound, two adjacent molecules accommodate with antiparallel orientation, giving rise to a more disordered columnar phase with respect to the phase formed by the non-polar molecules, showing instead parallel orientation and a more intense interaction among the cores. In contrast, there are examples in which the addition of polar substituents can generate more ordered mesophases, by the formation of 2D superlattices. Zhang and coworkers observed that by replacing an ether chain with an ester one in a HAT5 molecule it is possible to induce the formation of a 2D superlattice, because of interaction among the dipoles present on the modified molecules [171]. The model proposed to describe the superlattice formation is the following: inside each column, HAT5 modified molecules are in the more stable configuration when they are arranged in a way that leads to a net dipole component in the plane perpendicular to the columnar axis. To cancel this overall dipole, three columns arrange in triangular aggregates that form a hexagonal lattice and also a long-range ordered hexagonal 2D superlattice (Figure 22). The more ordered phases so obtained show ambipolar conductivity with both hole and electron mobilities slightly enhanced with respect to those of the unmodified HAT5. The same group studied the effect of changing two ether chains of HAT5 with pivalate groups, inducing the formation not only of a 2D superlattice, but also of monodisperse nanoparticles with a diameter of about 50 nm, as observed via transmission electron microscopy. In this case, the mobility increased by one order of magnitude [172].

A different approach used to improve mesophase order was proposed by Mu and coworkers. They prepared a series of side-chain polyacrylate polymers based on butoxy-substituted triphenylene (TP), studying their properties as a function of the length of the linking chain and of the degree of polymerization. They found that the polymers with shorter linkers and higher polymerization degree showed higher charge mobilities, comparable to those measured on hexahexylthio-substituted triphenylene-based DLCs. This was explained considering that the polymer linked structure inhibits dynamic disorder, boosting charge transport [173]. 

**Figure 23:**
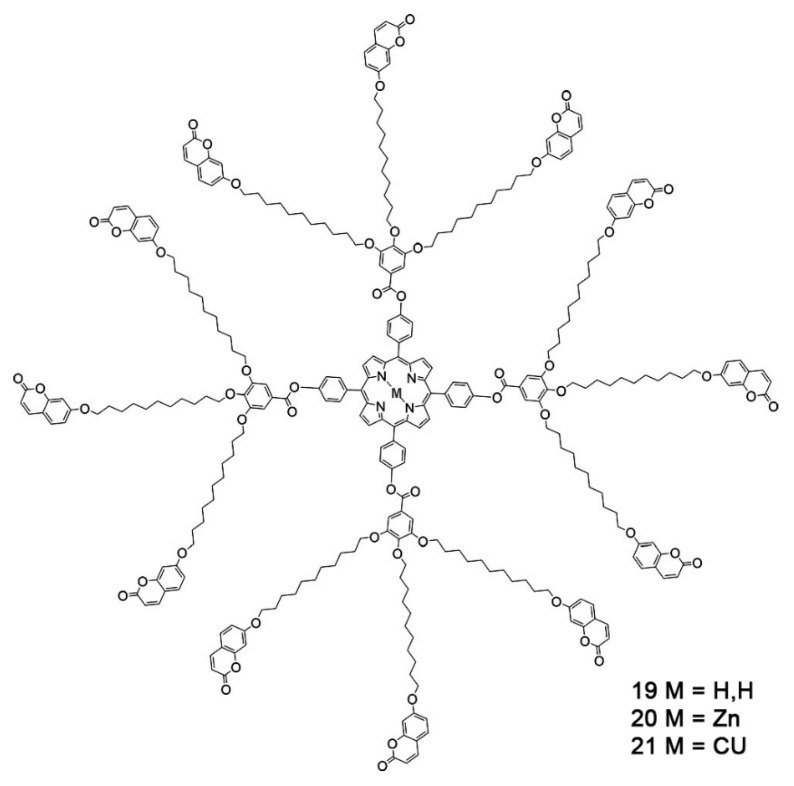
Porphyrin-core dendrimers with peripheral coumarin functional groups (adapted from reference [174] with permission of John Wiley and Sons. Copyright 2016 by John Wiley and Sons).

In a completely different approach, Concellòn and coworkers studied the mesomorphic and charge transport properties of porphyrin-core dendrimers with peripheral coumarin functional groups linked via alkyl chains (Figure 23). The change of paradigm introduced by such studies lies in the fact that these compounds form nematic discotic mesophases at room temperature instead of columnar phases. One first advantage of the nematic phase, in comparison with columnar ones, is a much lower density of structural defects. A second advantage of these particular compounds is a high tendency towards homeotropic alignment. Both features contribute towards the simple fabrication of devices with a homeotropic orientation, homogeneous on a scale of centimeters, even in 10 μm thick layers. Zn(II), Cu(II) and metal-free porphyrin derivatives were prepared. The hole mobility measured by SCLC was in the 0.1–1 cm^2^·V^−1^·s^−1^ range for the metal containing dendrimers, while it became 6–7 orders of magnitude lower for the porphyrins without metal. X-ray studies show no difference in structure between the mesophases formed by metal-free and metal-containing compounds. The role played by the metal in the enormous increase in mobility is unclear. Possible explanations include a direct involvement of the metal in the charge transfer process, a variation of the geometry of the porphyrin core leading to different transfer integrals or a metal-induced variation of short-range molecular order. The measured mobility values are the highest measured in nematic discotic phases and comparable to what is usually measured in the more ordered columnar mesophases [174]. The easy alignment and high mobility make this class of compound very promising for organic electronic applications [175,176].

### 5.2. Computational Approaches

Molecular modelling is a very powerful tool for predicting and understanding molecular behavior, through theoretical and computational approaches in many fields of chemical, physical and material sciences. In particular, in the field of DLC it has been used mainly for two different purposes: to guide towards the design of new molecules with improved properties and to understand the relationship between the chemical structure and the properties of DLC [177]. Different methods are usually employed and the results obtained are often complementary. In molecular mechanics (MM) approaches, atoms are described as point charges with an associated mass and the classical Newton mechanics laws are used to describe their interaction through van der Waals forces and electrostatic interactions. Molecular dynamics (MD) is used to add a time dependence, considering a system of interacting particles, setting and numerically solving the Newton’s motion equations. Equilibrium statistical mechanics, instead, is at the base of the Monte-Carlo method (MC) that involves the generation of states through Boltzmann statistics. A completely different approach is offered by quantum mechanics (QM) that is based on the solution of the Schrödinger equation to obtain the electronic structure of molecules. Within this frame, the molecular orbital theory can give information on electron density distribution within molecules. In the field of DLC, it is often used to calculate the energy and the distribution of the so-called frontier orbitals, HOMO and LUMO, since they are usually involved in charge transport processes. Even if the Hartree–Fock method can approximate the molecular orbital parameters, DFT is more commonly used, since it is usually less demanding in terms of computational power.

**Figure 24:**
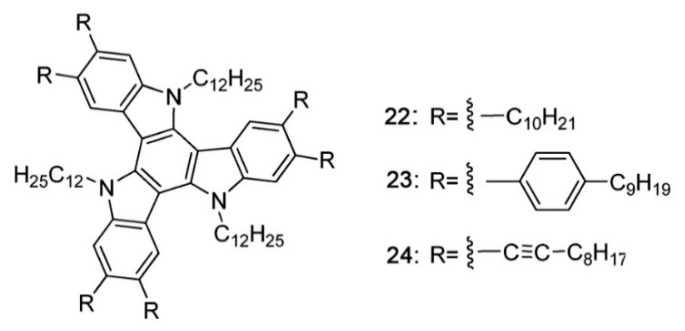
Molecular structure of the triindoles studied in reference [178]. (Adapted from reference [178], published by the PCCP Owner Societies).

As already mentioned, the application of different computational methods can provide complementary data that can lead to a complete interpretation of the studied phenomena. An example of how this approach can be applied in the field of DLC can be found in [178], where Volpi and coworkers report the studies on molecular electronic distribution and other molecular parameters that influence the mobility in the columnar phases of three triindole derivatives with very high hole mobility [178]. In particular, they studied a series of three triindoles with peripheral alkyl chains linked directly (**22**), or via phenyl (**23**) or alkynyl (**24**) linkers, to the core (Figure 24). As a first step, they used a QM approach to obtain molecular geometry, molecular orbital distribution and associated reorganization energy to transfer from neutral molecules to the respective radical cations. It was observed that the HOMO of compounds **22** and **23** is mainly located on the aromatic core, while in the case of **24** it extends over the alkynyl groups. Since low reorganization energies are usually related to higher mobility, it is also interesting to observe that the calculated reorganization energy of **24** is the lowest among the three compounds. In addition, MD studies revealed that mesophases of **24** show the most locked geometry, with a rotation of 60° between two successive molecules. This brings to more order columns and better π-orbital overlap and therefore higher mobility. These findings fit well with experimental data, since compound **24** showed the highest mobility.

**Figure 25:**
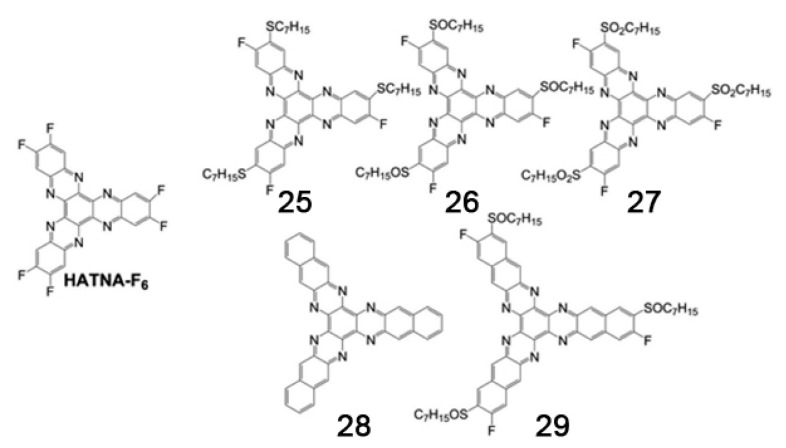
Chemical structures of hexaazatrinaphthylene-based discotic mesogens (adapted from reference [180] with permission of John Wiley and Sons. Copyright 2017 by John Wiley and Sons).

An and coworkers, studied the properties of a series of five hexaazatrinaphthylene-based discotic mesogens (Figure 25) based on HATNA-F6. Compounds **25**, **26** and **27**, obtained replacing three F atoms with three alkylsulfanyl chains (sulfide for compound **25**, sulfoxide for compound **26** and sulfone for compound **27**), had been already used as efficient electron-transporting materials (ETMs) for perovskites solar cells [179], while compounds **28** and **29** were only theoretically designed [180]. Frontier orbitals and reorganization energies, for both hole and electron transport, were obtained via DFT, while charge mobilities were calculated from MD and semiclassical Marcus charge-transfer theory. HOMO and LUMO energies and electron mobilities calculated for compounds **25**–**27** are in good agreement with the experimental values. Compounds **28** and **29**, with extended π-conjugated cores, show lower reorganization energies than compounds **25**–**27**, for both electron and hole transfer. Moreover, compound **29** shows the highest transfer integrals and calculated charge mobilities for both holes and electrons and, therefore, it can be considered as a potential candidate to be used in optoelectronics applications.

Among the most studied compounds in the field of DLC are triphenylene derivatives, not only from the experimental point of view but also considering modelling [181,182]. Thompson and coworkers proposed an innovative analysis that easily provides the connection between microscopic properties and performance, investigating the relationship between molecular order and charge transport at the mesoscale, by analyzing a volume of around 20 × 20 × 60 nm^3^ containing more than 9000 molecules of the hexa-octyl-thio-triphenylene discotic mesogen [182]. Both ordered columnar and disordered morphologies were modelled via MD, charge mobility was calculated through MC methods and the rapid increase of mobility upon switching from disordered to ordered morphology was simulated. They also used graph theory to analyze the charge trajectories within the volume, considered as a transporting network, and to establish a correlation between mobility and morphology. In this way, it was possible to identify pairs of molecules characterized by very high transfer rate and to conclude that pairs must be suitably spatially correlated otherwise they can act as traps, lowering charge mobility.

**Figure 26:**
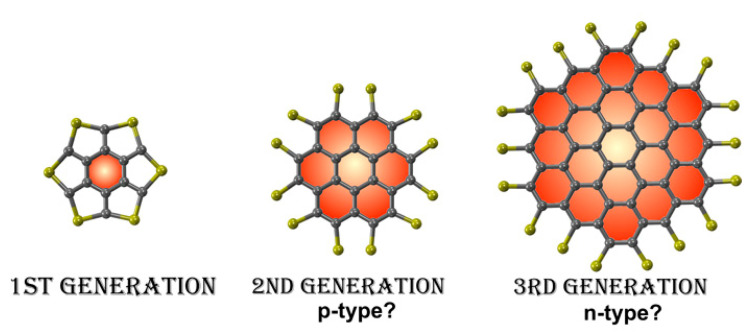
Schematic representation of the Selflower and Sulflower molecular structures, with S or Se atoms in yellow. (Adapted with permission from reference [183]. Copyright 2017 American Chemical Society).

The group of V. Lukeš focused its attention on theoretically designed π−conjugated cores to find which ones are the best candidates to synthetize new compounds with improved properties for applications in optoelectronic devices [183,184]. In one work [183], they considered a series of 43 derivatives of the heterocyclic circulenes, called Sulflowers if the heteroatoms are sulfur atoms, or Selflowers if selenium atoms are present. Figure 26 is a schematic representation of the molecular structures investigated. As a first step, DFT was used to optimize the geometry of neutral and charged states, to calculate the HOMO and LUMO energies and to analyze the reorganization energies. Marcus theory was then used to calculate drift mobilities. On the basis of the reorganization energies and charge transport characteristics, the second-generation compounds show properties suitable for p-type transport, for both Sul- and Selflowers, while the third generation of Sulflowers was predicted to be suitable to build ambipolar semiconductors with the highest drift mobility.

### 5.3. Mesophase Alignment

**Figure 27:**
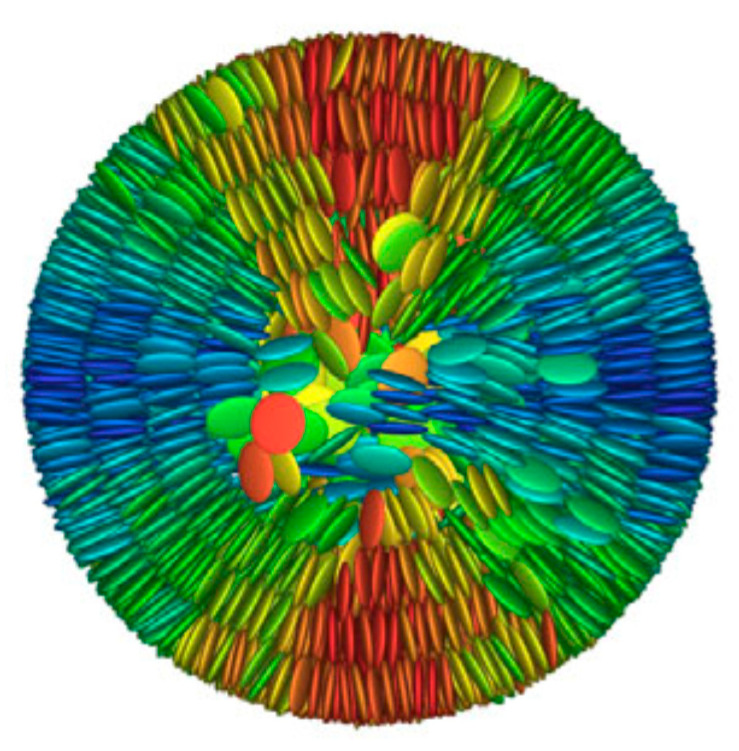
A Monte Carlo simulation snapshot illustrating the morphology of hexakis(n-hexyloxy)triphenylene (HAT6) discotic liquid crystals (DLC) in 17 nm pore (reprinted with permission from reference [190]. Copyright 2018 American Physical Society).

As already underlined, one of the most important and peculiar characteristics of DLC is their one-dimensional charge mobility. In order to exploit this property, it is necessary to align all columns along the same direction otherwise, except for some very rare cases [185], the effective mobility is much lower, even by several orders of magnitude [186]. This alignment process is complex, since DLC are usually characterized by a very high viscosity, that makes it hard to exploit interactions with electric and magnetic fields or with surfaces, as commonly done with calamitic liquid crystals to obtain monodomains. For this reason, a consistent amount of work was dedicated to the task of obtaining well-aligned mesophases. Zhao and coworkers demonstrated [187] that it is possible to obtain a large area homeotropic alignment of an anthraquinone derivative, a n-type mesogen, on a single substrate by cooling from the isotropic phase to room temperature at a rate of 6 °C per hour, obtaining single-domain sizes of the order of millimeters. The homeotropic alignment not only leads to a much stronger optical absorption, due to the ordered π−π stacking and favorable orientation of aromatic rings, but also to an increase of electron mobility up to 1.2 × 10^−2^ cm^2^·V^−1^·s^−1^, about 2 orders of magnitude higher than the value measured in randomly crystallized films. Gujral and coworkers [31] studied the parameters that can influence the morphology of films of two DLC obtained via vapor deposition of 2,6,10-triethoxycarbonyl-triphenylene (a rectangular columnar mesogen) and 1,16-di(methoxycarbonyl)-6,7,12,13-tetra(ethoxycarbonyl)-phenanthro[ghi-1,2,3,4]perylene (a hexagonal columnar mesogen). On a first heating, both compounds show a transition from a crystalline to a columnar phase, while on cooling from the isotropic phase, they form columnar phases that are metastable against crystallization up to room temperature. The depositions were performed using either a silicon ⟨100⟩ substrate with native oxide or a fused silica substrate, at temperatures ranging from 0.75 Tg up to 0.99 Tg during the deposition. For both materials, Tg is ~100 °C lower than the transition from crystalline to liquid crystals. Characterizations of the resulting films by grazing incidence wide-angle X-ray scattering clearly showed that when the substrate temperature is around Tg, disordered glassy states are obtained, while at substrate temperatures around 0.75 Tg, homeotropic alignment is obtained. A different approach was used by Kondratenko and coworkers [188], who studied three different composites obtained adding respectively 1%, 3% and 5% of the crosslinkable nematogen 2-methyl-1,4-phenylene bis(4-((6-(acryloyloxy)hexyl) oxy) benzoate) (RM) to the well-known hexagonal columnar mesogen 2,3,6,7,10,11-hexapentyloxytriphenylene (HAT5). They prepared samples in which RM was aligned by the interaction with a rubbed polyimide layer and then polymerized by ultraviolet (UV) light. In this way, HAT5 resulted better aligned and the hole mobility measured by TOF increased by about 30% for samples with 1% and 3% of RM with respect to the mobility of pure HAT5 [16]. However, the HAT5 morphology was disturbed by too high an amount of RM and at 5% of RM the increase of mobility was only 10%. The mechanism that leads to a better orientation of the HAT5 is not clear. Another interesting approach from the applicative point of view was proposed by Park and coworkers [189], who studied the orientation of an ambipolar DLC formed by donor-acceptor supramolecules obtained via H-bonding between a star-shaped tris(triazolyl) triazine acceptor and three triphenylene-derived donors. The mesophase was inserted in a one-dimensional porous anodic aluminum oxide (AAO) film. The mesophase orientation was controlled by both pore size and chemical modifications of the inner surface of the pores. In pores with 100 nm diameter, the molecular stacks tend to align perpendicularly to the pore axis, with the molecules flat on the walls of the pores. In pores of 20 nm diameter, probably because of the higher curvature, the molecular stacks tend to align parallel to the pore axis. If the chemical affinity of the surfaces of the pores is changed, by adding tridecafluoro-1,1,2,2-tetrahydrooctyltrichlorosilane, axially oriented columns can be observed in the 100 nm pores as well. A different morphology was found for DLC formed by hexakis(n-hexyloxy)triphenylene (HAT6) within the 17 nm pores of a silica membrane [190]. In this case, decreasing the temperature from the isotropic phase, the discotic molecules closest to the pore wall start to orient with their short axes parallel to the walls and normal to the pore axis, forming a bent columnar ring. At lower temperatures, also the inner molecules start to form up to 5 bent columns, concentric with the previous one. (Figure 27). A comprehensive review of alignment methods can be found in reference [33].

### 5.4. Synthetic Developments

In this subsection, the last developments regarding the synthesis of novel discotic mesogenic materials will be described. Triphenylene derivatives still remain the most studied compounds and they have been used to develop new materials, testing new side chains, or new cores [191,192,193,194,195,196], to be linked with other cores to prepare dyads and triads [67,197,198,199,200] or materials giving segregate columns [201,202]. Triphenylenes were also doped with nanoparticles or used to prepare blends with other mesogenic molecules [203,204,205].

**Figure 28:**
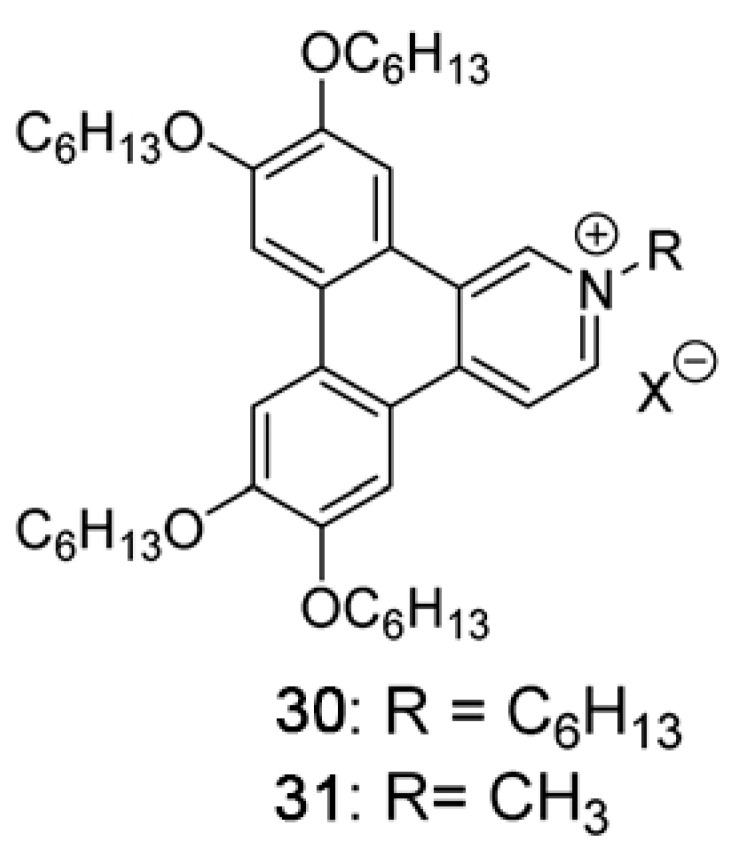
Molecular structure of azatriphenylene-based DLC.

**Figure 29:**
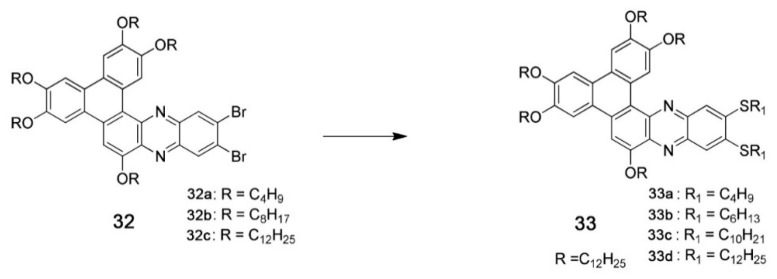
Molecular structures of the phenazine fused triphenylenes studied in reference [193]. (Adapted from [193] with permission of John Wiley and Sons. Copyright 2018 by John Wiley and Sons).

One example of studies on modified triphenylenes was provided by Zhao and coworkers, who developed a series of 15 non-symmetrical triphenylenes derivatives, adding both electron-withdrawing and electron-donating groups. They observed that only compounds bearing fluorine, chlorine and carbazole side chains form columnar mesophases [194]. The compounds with halogen atoms show an increase of charge mobility, while the carbazole derivative shows lower mobility, when compared to their symmetric triphenylene homologues. The presence of carbazole perturbs the order of the mesophase because its intrusion among the disks disturbs the stacking, making charge transfer less effective. The presence of halogen substituents disturbs the stacking as well, but in this case the electrostatic attraction due to the dipole–dipole interaction can balance such effect and the mobility is higher than in the symmetric hexa(alkoxy)triphenylenes. More recently, the same group studied a series of ionic compounds based on azatriphenylenes, a triphenylene derivative in which a carbon atom has been replaced by nitrogen in the aromatic core (Figure 28). Almost all the synthetized compounds formed columnar mesophases and, in some cases, also the counterions show columnar arrangement [194]. The group of S. Kumar focused the attention on the study of a novel core based on π-extended phenazine-fused triphenylene, synthesizing compounds with different substituents and evaluating the effect they have on mesophase properties [191,192,193]. The first system proposed was a phenazine fused triphenylene with alkanethiols side chains (Figure 29): mesomorphic properties were studied for compounds **33 a–d**, as well as for the di-bromine intermediates **32 a–c** [193]. All compounds form hexagonal columnar phases, with the thiol compounds showing the mesophases at lower temperatures, and DFT studies showed that HOMO and LUMO orbitals are delocalized also on the phenazine portion of the core. Charge mobility was measured for compound **33 b** and it is of the order of 10^−4^ cm^2^·V^−1^·s^−1^. The same core was used to prepare four more derivatives, adding an ester group to the end of the phenazine portion [191]. Even in this case, the four synthetized compounds form hexagonal columnar mesophases over a broad temperature range and are stable at room temperature. Interestingly, the compounds showed also non-linear optical properties that make them suitable for optoelectronic applications. Another set of compounds based on the phenazine fused triphenylene core was developed adding alkyl and alkoxy phenylacetylene chains on the phenazine portion of the core. Also, such compounds form columnar mesophases stable at room temperature and exhibit non-linear optical properties [192].

**Figure 30:**
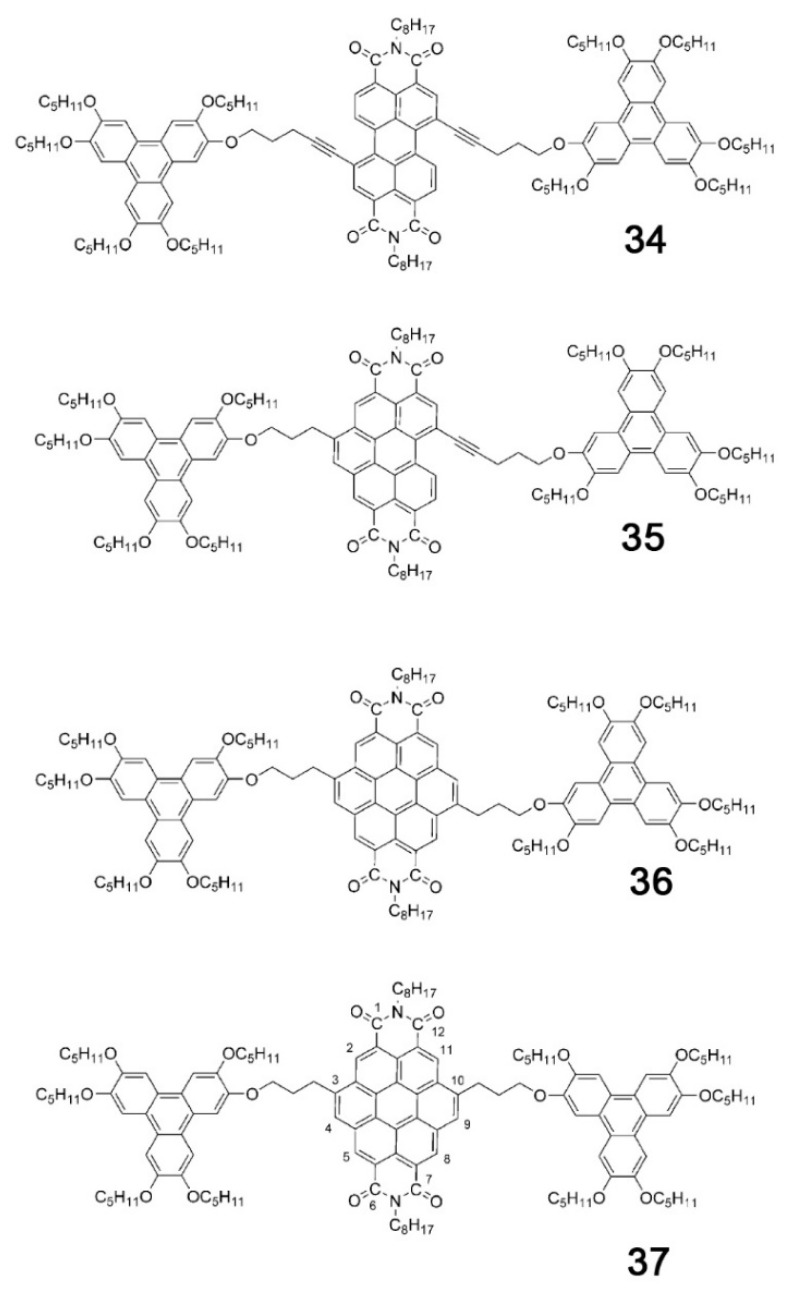
Molecular structures of triads studied in reference [206].

Triphenylene was also used as a constituent of dyads and triads, often in molecular system in which an electron donor (D) is covalently linked to an electron acceptor (A), with the aim of obtaining a “molecular heterojunction” [168,197,198,200,206,207]. This kind of molecular system can be a good candidate to produce optoelectronic devices, such as organic light-emitting diodes (OLED) and organic solar cells (OSC). If the system is well designed, the donor and the acceptor can form segregated columns, providing distinct pathways for separated positive and negative charges, a feature that can improve the properties of devices when charge recombination is to be avoided. Zhao and coworkers prepared a series of four triads based on two triphenylene mesogens, acting as electrons donors, laterally linked to a central benzoperylenediimide, coronenediimide or perylenediimide group that behaves as the electron acceptor (Figure 30).

**Figure 31:**
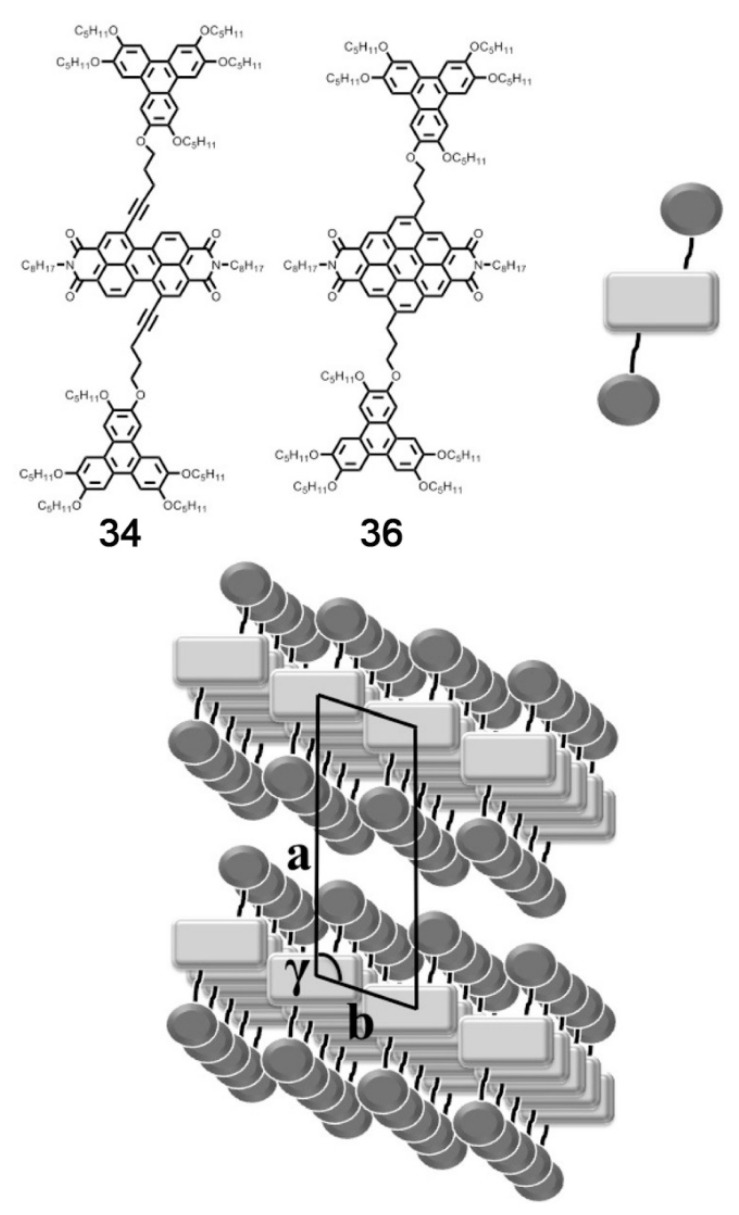
Molecular structures and model proposed for the mesophases formed by compounds **29–32**. a, b, and γ are crystallographic parameters (adapted from reference [206] with permission of John Wiley and Sons. Copyright 2015 by John Wiley and Sons).

Photophysical experiments showed a complete fluorescence quenching in all four triads, due to the efficient charge transfer of the photo-generated charges between the triphenylene and the central unit, demonstrating that the triads can work as molecular heterojunction. POM, DSC, and SAXS revealed that the triads form lamello-columnar oblique mesophases over a wide range of temperature, with the triphenylene and the central units segregated in well separated columnar stacks insulated by the aliphatic chains (Figure 31). The measured TOF mobility was 2.6 × 10^−2^ cm^2^·V^−1^·s^−1^ for electrons and 1.8 × 10^−3^ cm^2^·V^−1^·s^−1^ for holes [206]. Another example of triphenylene-based dyads was provided by Kong and coworkers, who prepared a series of dyads linking a hexa(alkoxy)triphenylene with a perylene tetracarboxylic ester, with alkyl chains of different lengths. They observed the formation of columnar mesophases in which the electrochemical behavior of donors and acceptors is preserved. Photophysical properties were also studied and emission quenching observed for all the synthetized compounds. The emission intensity varies with the length of the linking chains: it is almost completely quenched for dyads linked by the shortest chain, while it gradually increases with the increase of the length of the linking chains [197].

**Figure 32:**
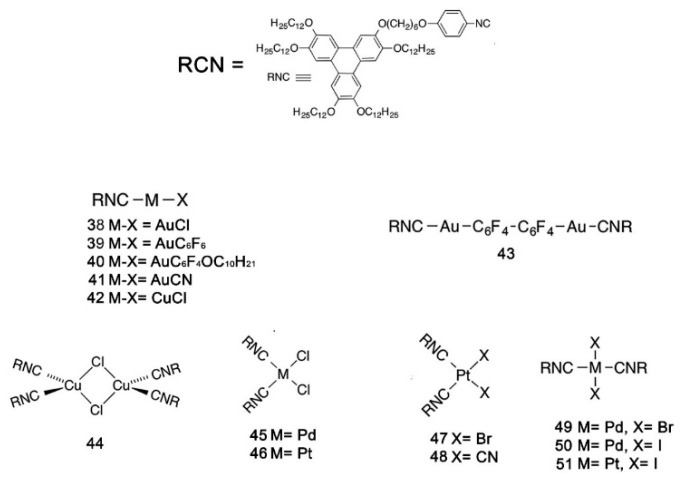
Isocyano-triphenylene gold, copper, palladium, and platinum complexes forming segregate columns.

Segregation is observed not only in triads or dyads, but also in other molecular systems and it can be used to improve the properties of DLC. The group of Espinet reported the formation of DCL from a series of isocyano-triphenylene gold, copper, palladium, and platinum complexes bearing up to four triphenylene units (Figure 32). The formation of rectangular columnar mesophases was observed in all cases, except for the Au-Au compounds, as well as the nano-segregation of the triphenylene columns from the columns containing metal moieties. Even if the π–π stacking of triphenylene fragments is a fundamental driving force for mesophase formation, the role of the metal fragments can be also very important and it can contribute to mesophase formation via the interaction of the high dipole moments deriving from the presence of the metal. The models proposed for supramolecular organization show that mesophase formation for compounds with a high number of triphenylenes per molecule is favored by the enthalpic stabilization given by the π−π interaction of triphenylenes, while in molecules with only one triphenylene fragment, mesophase formation is favored by the interaction of the high dipole moments deriving from the presence of the metal. Hole mobility depends on mesophase alignment, reaching values up to 10 cm^2^·V^−1^·s^−1^ with optimal alignment, among the highest values measured in triphenylene mesogens [201]. The same group studied the mesomorphic properties of (i) imidazolium salts containing one or two triphenylene units linked through a hexyloxy chain, with metal-complexes as counterions; and (ii) related triphenylene–carbene–metal complexes [202]. They observed the formation of hexagonal columnar mesophases, with segregated columns of triphenylene and imidazolium moieties. Also in this case, the phase formation driving force is the π–π stacking of triphenylene cores, with the ionic and the dipole–dipole interactions giving an essential contribution as well.

**Figure 33:**
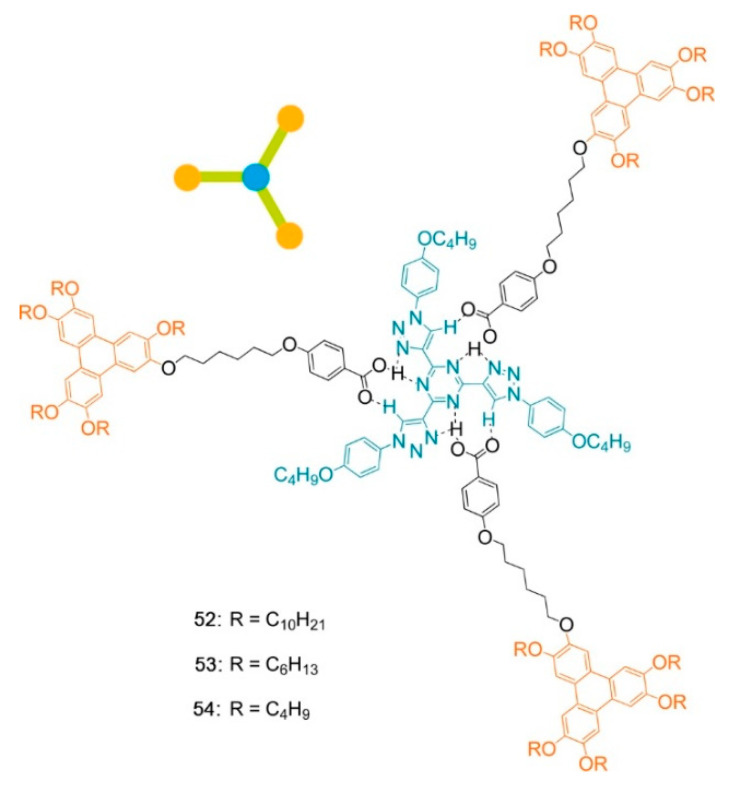
Molecular structures of hydrogen bonded compounds based on tris(triazolyl)triazine and triphenylene containing acids, forming segregated columns (adapted with permission from reference [199]. Copyright 2018 American Chemical Society).

Feringán and coworkers reported instead about the synthesis of ambipolar mesomorphic hydrogen-bonded molecules based on tris(triazolyl)triazine and triphenylene containing acids, forming segregated columns (Figure 33). Although the single components do not form columnar mesophases, the complexes obtained through hydrogen bond form hexagonal columnar mesophases with the triphenylene and triazine columns segregated from each other, as shown by X-ray diffraction. The synthesized complexes show ambipolar charge mobility with an electron mobility in the 10^−2^–10^−1^ cm^2^·V^−1^·s^−1^ range and hole mobility around 10^−3^–10^−2^ cm^2^·V^−1^·s^−1^ [199].

Triphenylenes were also doped with different kinds of molecule to improve charge mobility or electric conductivity [203,204,205]. Khan reported results on a blend of HAT6 and 2,3,6,7,10,11-hexakisdecyloxytriphenylene (HAT10) doped with different amounts (from 0.1 to 5 wt%) of 2,3,5,6-tetrafluoro-7,7,8,8-tetracyanoquinodimethane (F4TCNQ), demonstrating an increase of conductivity due to doping [205]. Shah and coworkers reported instead on the doping of a phenazine-fused triphenylene with oleylamine-capped CdS nanowires. Even if they observed a decrease in the core-core distance in the doped mesophase, a small decrease of hole mobility was detected and attributed to the effect of the nanowires on DLC order. However, an increase of electrical conductivity was observed because the CdS nanowires can behave as an n-type dopant [204]. Mahesh and coworkers studied the effect of doping 2,3,6,7,10,11-hexabutyloxytriphenylene (HAT4) with different concentrations (from 0.5 to 5 wt%) of carbon nanodots. As observed by POM, carbon nanodots were uniformly dispersed, and small-angle X-ray diffraction and differential scanning calorimetry revealed that they did not perturb the mesophase order. The core-core distance in this case was not affected, and the observed decrease of charge mobility was attributed to the interaction between the aromatic system of HAT4 and the sp^2^ orbital system of the carbon nanodots [203].

Besides triphenylene, other molecular systems were studied in recent years, such as those based on triindole [208,209], phthalocyanine [125,210], thiophene [211,212] and truxenone [213,214] cores, or on particular molecular architectures, such as dendrons [174,176,215,216]. Nakawa and coworkers reported the study of the mesomorphic and mobility properties of a blend of 1,4,8,11,15,18,22,25-octahexylphthalocyanine (C6PcH2) with its analogue 1,4,8,11,15,18,22,25-octahexyltetrabenzotriazaporphyrin (C6TBTAPH2) [125]. The interest on DLC blends arises from the evidence that they can improve the properties of optoelectronic devices, such as for example OSC, in which the use of a blend of DLC’s as the active layer led to an improvement of efficiency and short circuit current by about 30% with respect to the cells prepared with a single DCL [217]. The effect is quite interesting, although its origin is still unclear. From POM observations, crystal structure analysis and DSC measurements it was inferred that the two compounds are miscible at all concentration in mesophases, while they are not in the crystalline phase. The mobility values are 0.5 cm^2^·V^−1^·s^−1^ in the hexagonal discotic phase and 0.2 cm^2^·V^−1^·s^−1^ in the crystalline phase [125].

**Figure 34:**
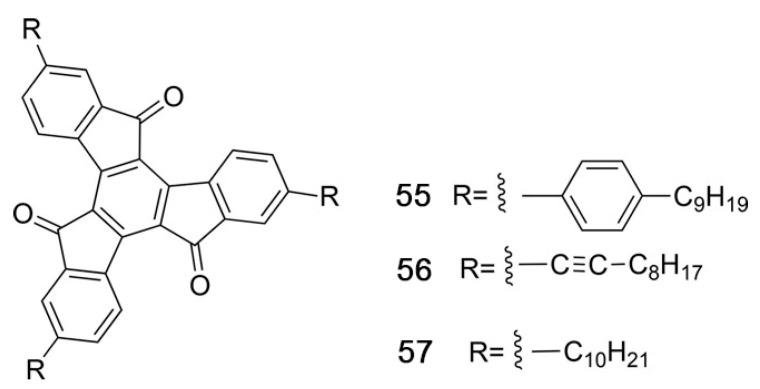
Molecular structures of truxenones (reprinted from reference [213] with permission of John Wiley and Sons Copyright 2018 by John Wiley and Sons).

DLC that show high electron conduction are not too common. An interesting example is represented by the truxenones derivatives studied by Gomez-Esteban and coworkers [213]. A SCLC mobility of about 1 cm^2^·V^−1^·s^−1^ was reported for compounds **55** and **56**, while it was two orders of magnitude lower for compound **57** (Figure 34). DFT and semiclassical Marcus theory were used to predict mobility values that are very close to the measured mobility for compounds **55** and **56**, while the same agreement was not found in the case of **57**. The lower mobility measured in **57** and the difference with the theoretically predicted mobility cannot be explained in terms of intrinsic molecular factors and, therefore, they were attributed to a decrease in intra-stack order when alkyl chains are linked directly to the core [213].

Ambipolar compounds were widely studied because they can be used in several applications. An example of DLC with ambipolar conduction was provided by Liu and coworkers, who studied a series of benzothienobenzothiophenes cored columnar mesogens. All compounds formed hexagonal columnar mesophases over a broad temperature range. Charge mobility for both electrons and holes ranges around 10^−3^ cm^2^·V^−1^·s^−1^ [211].

### 5.5. Applications of Discotic Liquid Crystals (DLC)

**Figure 35:**
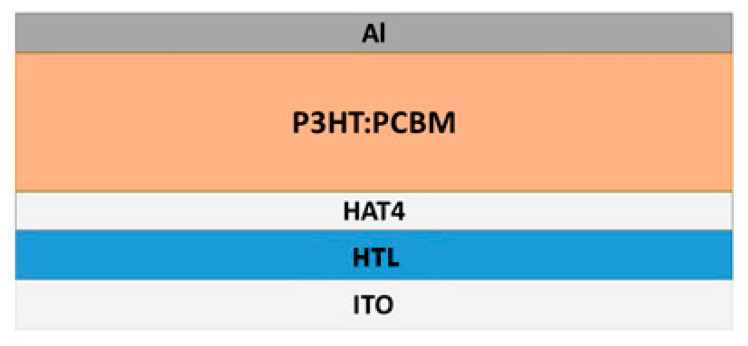
Schematic representation of bulk-heterojunction cell with a 2,3,6,7,10,11-hexabutyloxytriphenylene (HAT4) interlayer.

DLC have been widely used to prepare OSCs, OLEDs and OFETs. The first observation of the photovoltaic effect in DLC was reported by Gregg et al. [218], who observed the photovoltaic properties of a phthalocyanine derivative in symmetric cells. A review of the results obtained using DLC in the field of OSC can be found in reference [219], while the newest results will be presented here. Bajpai and coworkers studied how the presence of a layer of the 2,3,6,7,10,11-hexabutyloxytriphenylene mesogen can influence the performance of the classic bulk-heterojunction OSC formed by poly (3-hexylthiophene) and [6,6]-phenyl-C61-butyric acid methyl ester (P3HT:PCBM) [220,221]. They added the DLC layer between the hole extracting layer and the bulk heterojunction, studying the effect on two different devices (Figure 35): in the first one, PEDOT:PSS was used as hole acceptor, while in the second one the hole acceptor was MoO_3_. In both cases, an increase of photovoltaic performances was observed respect to the devices without any DLC layer, with an increase of more than 100% of power conversion efficiency, mainly due to an increase of short-circuit current. In both cases it was important to perform an annealing process to induce the homeotropic alignment of the mesophase. The same group reported results in a similar system, in which a layer of HAT4 was included between the MoO_3_ hole extracting layer and a bulk heterojunction with [6,6]-phenyl C_71_-butyric acid methyl ester (PC_71_BM) instead of PCBM [222]. In this case, an increase of power conversion efficiency of about 70%, due to the increase of short-circuit current was observed. Ozaki et al. described the use of the mesogenic 1,4,8,11,15,18,22,25-octahexylphthalocyanine (C6PcH2) compound to prepare OSC. In a first study, they reported results on a C_60_/C6PcH2 bilayer, compared with a PCBM/C6PcH2 bulk-heterojunction [223]. In a second work, investigations were extended to the same bulk-heterojunction, in which the alignment of the DLC was improved by the effect of a poly(vinyl phenol) layer [146]. In both cases, results suggest that in the bulk-heterojunction devices the improvement of alignment leads to higher efficiency. Bulk-heterojunctions prepared using DLC were also used to prepare tandem OSC, coupling a standard bulk-heterojunction cell based on P3HT/PCBM with a cell formed by the bulk-heterojunction C6PcH2/PCBM [224]. Ozaki and coworkers also used another approach, preparing a Bulk-Heterojunction cell by mixing PCBM with a blend of 2 DLC: C6PcH2 and its homologue, 1,4,8,11,15,18,22,25-octapentylphthalocyanine (C5PcH2), because it is well known that in this way it is possible to obtain DLC mesophases with higher mobility. The power conversion efficiency increased by about 10% in the blend in which 25 mol% of C6PcH2 is present, with respect to the cell containing C5PcH2 only. More details on the studies of OSC prepared using phthalocyanine-based liquid crystals can be found in reference [225].

**Figure 36:**
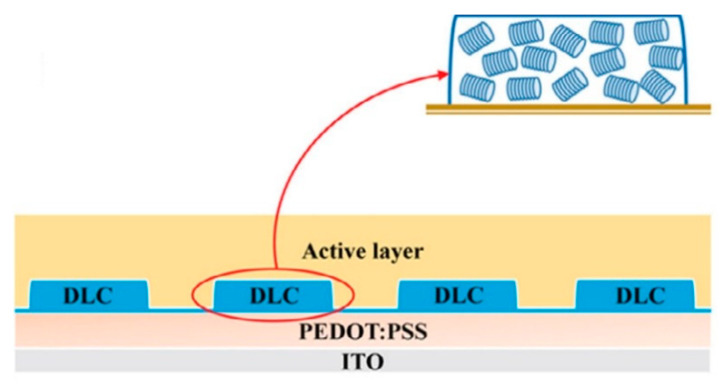
Columns formed by 2,3,6,7,10,11-hexaacetoxytriphenylene (HATP) on PEDOT:PSS and column orientation within the mesophase. (Reprinted from reference [226] with permission of John Wiley and Sons. Copyright by 2020 John Wiley and Sons).

Recently, Wang and coworkers published a paper in which the effect of the addition of a layer of the commercial 2,3,6,7,10,11-hexaacetoxytriphenylene (HATP) mesogen between PEDOT:PSS and the active layer of a bulk-heterojunction cell is described [226]. The DLC was deposited on PEDOT: PSS by spin-coating and it formed macrodomains with an edge-on orientation of the columns, surrounded by the active layer, as shown in Figure 36. The high mobility of HATP helped charge migration within the active layer, increasing the short-circuit current and consequently the power conversion efficiency by about 10%.

**Figure 37:**
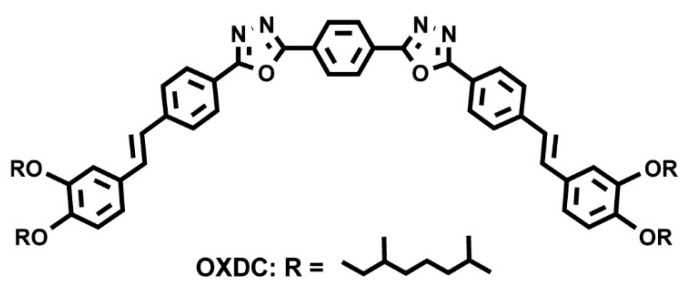
Molecular structure of OXDC (reprinted with permission from reference [228]. Copyright 2017 American Chemical Society).

The high one-dimensional mobility is a key factor that makes DLC attractive for developing innovative OLED’s. For this reason, research in the field of emissive DLC has been rapidly increasing in the last years and the number of reports on OLED devices based on emissive DLC is now growing [227]. Sivadas and coworkers synthetized a novel chiral molecule called OXDC (Figure 37) that gives rise to a columnar helical phase. Results from temperature-dependent wide-angle X-ray diffraction (XRD) investigations, confirmed that the discotic cores are tilted with respect to the columnar axis, forming a helical structure that stabilizes the phase. The material was used to prepare an OLED with the configuration ITO/PEDOT:PSS/OXDC/Al characterized by a luminescence of 10,115 cd·m^−2^ [228].

**Figure 38:**
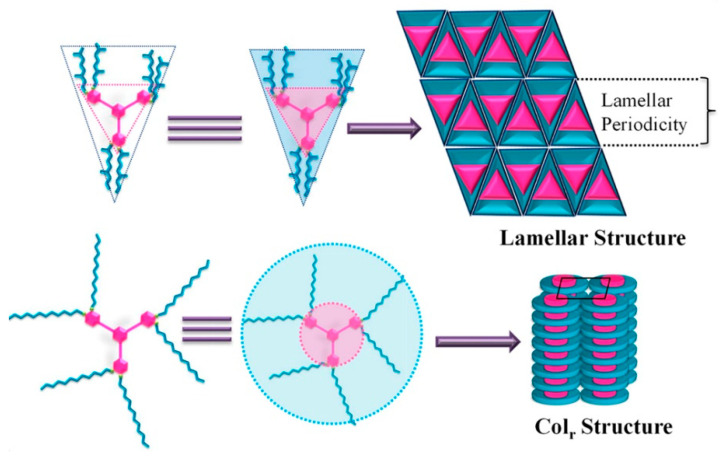
Representation of the phase structures formed by emitting DLC based on oligo(phenylenevinylene) cores (reprinted with permission from reference [229] Copyright 2018 American Chemical Society).

De and coworkers studied instead the mesomorphic and emissive properties of newly synthetized compounds based on an oligo(phenylenevinylene) core, known for its emission in the blue, linked to six alkoxy chains. They synthetized compounds with different lengths of the alkoxy chains, that formed lamellar phases in the case of shorter chains and columnar rectangular phases in the case of longer chains (Figure 38). One of the compounds was used as the emissive layer in an OLED with the architecture ITO/PEDOT:PSS/emitting layer/TPBi/LiF/Al, where TPBi stands for 2,2′,2′’-(1,3,5-benzinetriyl)-tris(1-phenyl-1-H-benzimidazole), measuring a luminance of 450 cd·m^−2^ at 10 V [229].

**Figure 39:**
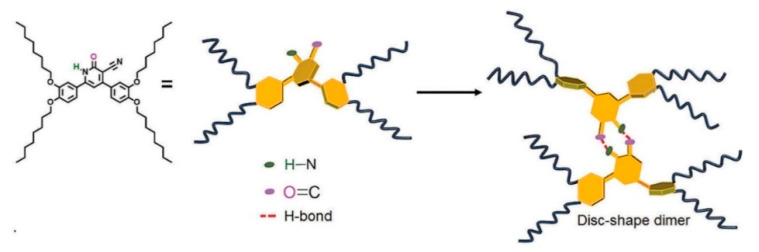
Representation of H-bonded disk-like dimers in donor-acceptor molecules (reprinted with permission from reference [232], published by Royal Society of Chemistry. Copyright 2016 by Royal Society of Chemistry.

Bala and coworkers reported the performance of an OLED built using a DLC formed by a blue emissive s-heptazine discotic core linked to tri-alkoxy benzene rings. They observed that the emission is higher in the solid state, rather than in solution. This is probably due to the spatial separation of LUMO and HOMO, as demonstrated by DFT studies revealing that the HOMO is located on the tri-alkoxy benzene rings while the LUMO is located on the core. The location of HOMO and LUMO in two separated molecular portions can provide separate transport channels for electrons and holes and this can increase emission efficiency. Emissive properties were tested with an OLED with the following architecture: ITO/PEDOT:PSS/Host:Emitter/TPBi/LiF/Al where the emitter based on s-heptazine was added at different dopant concentrations. Several different hosts were used and the best performances were obtained using 4,4′-bis(9-carbazolyl)-1,1′-biphenyl (CBP) [230]. More recently, the same authors studied the emission of an OLED prepared using a perylene based columnar liquid crystal as an emitter, obtaining a luminescence of about 5520 cd·m^−2^ [231]. The separation between acceptor and donor fragments was used also by Vinayakumara and coworkers, who synthesized a novel mesogenic molecule with a D–A–D architecture based on a cyanopyridone derivative. XRD patterns show that the driving force for mesophases formation is the H-bonding between the lactame rings, leading to the formation of dimers that are the building blocks of the columnar phase (Figure 39). They prepared OLEDs with different architectures in which the DLC was used as an emitter in two different ways: pure or dissolved in poly (9-vinylcarbazole) (PVK). The best results were obtained using the blend, with a luminance of 1055 cd·m^−2^ [232]. More recently, the same group designed a new compound formed by the electron-rich 9,10-didodecyloxyphenanthrene and trialkoxybenzene cores as electron donors linked to the 3-cyanopyridone moiety as an electron acceptor. The electroluminescent properties were tested preparing OLEDs with the DLC used as a neat emitter or dissolved in PVK. The best results were reached in the last case, with a luminance of 1898 cd·m^−2^, while in the case of DLC used as a neat emitter a luminance of 258 cd·m^−2^ was reached [233].

As illustrated in Section 4, OFET are often used to measure charge mobility of DLC. Because of their one-dimensional mobility, DLC can be used to prepare high-performance OFET, as shown by Canimkurbey and coworkers, who developed an OFET based on a Cu-phthalocyanine bearing triple-branched alkylthio chains [234]. The same researchers also explored new properties, in order to implement innovative devices based on DLC, such as for example the lithium-ion conductivity in a hexaazatrinaphthylene-polyether (HATN-TEG-1) doped by polyethylene oxide (PEO). With doping up to 5% of PEO, the columnar organization was maintained and an ionic conductivity of 6.06 × 10^−7^ S·cm^−1^ was reached [235].

## Abbreviations

AAO	Anodic Aluminum Oxide
C6PcH2	1,4,8,11,15,18,22,25-octahexylphthalocyanine
C6TBTAPH2	1,4,8,11,15,18,22,25-octahexyltetrabenzotriazaporphyrin
CELIV	Charge Extraction by Linearly Increasing Voltage
Col_h_	Columnar Hexagonal
Col_obl_	Columnar Oblique
Col_r_	Columnar Rectangular
DFT	Density Functional Theory
DI-SCLC	Dark Injection Space Charge Limited Current
DLC	Discotic Liquid Crystals
DSC	Differential Scanning Calorimetry
ETM	Electron-Transporting Materials
F4TCNQ	2,3,5,6-Tetrafluoro-7,7,8,8-tetracyanoquinodimethane
GIWAXS	Grazing Incidence Wide-angle X-ray scattering
HAT3	2,3,6,7,10,11-hexapropoxytriphenylene
HAT4	2,3,6,7,10,11-hexabutyloxytriphenylene
HAT5	2,3,6,7,10,11-hexapentyloxytriphenylene
HAT6	2,3,6,7,10,11-hexakis(n-hexyloxy)triphenylene
HATN-TEG-	hexaazatrinaphthylene-polyether
HATP	2,3,6,7,10,11-hexaacetoxytriphenylene
HOMO	Highest Occupied Molecular Orbital
SAXS	Small-Angle X-ray Scattering
IUPAC	International Union of Pure and Applied Chemistry
CELIV	Charge Extraction by Linearly Increasing Voltage
ITO	Indium tin oxide
LUMO	Lowest Occupied Molecular Orbital
MC	Monte-Carlo
MD	Molecular Dynamics
MM	Molecular Mechanics
N_C_	Nematic Columnar
N_D_	Nematic Discotic
N_L_	Lateral Nematic
OFET	Organic Field-Effect Transistor
OLED	Organic Light Emitting Diode
OSC	Organic Solar Cell
P3HT	poly (3-hexylthiophene)
PC_71_BM	[6,6]-phenyl C_71_-butyric acid methyl ester
PCBM	[6,6]-phenyl-C_61_-butyric acid methyl ester
PEDOT: PSS	poly(3,4-ethylenedioxythiophene) polystyrene sulfonate
PEO	polyethylene oxide
POM	Polarized Optical Microscopy
PR-TRMC	Pulse-Radiolysis Time-Resolved Microwave Conductivity
PVK	poly (9-vinylcarbazole)
QM	Quantum Mechanics
RM	2-methyl-1,4-phenylene bis(4-((6-(acryloyloxy)hexyl) oxy) benzoate)
SAXS	Small Angle X-ray Scattering
SCLC	Space Charge Limited Current
TEG	Triethyleneglycol
TOF	Time of Flight
TPBi	2,2′,2″-(1,3,5-Benzinetriyl)-tris(1-phenyl-1-H-benzimidazole)
